# Matrix Metalloproteinases as Candidate Antigenic Determinants for Anti‐Tumor Autoantibodies in Human Ovarian Cancer: A Post Hoc Analysis

**DOI:** 10.1096/fj.202500739RR

**Published:** 2025-09-23

**Authors:** Christopher Markosian, Alexander M. Ille, Stephen K. Burley, Wadih Arap, Renata Pasqualini

**Affiliations:** ^1^ Rutgers Cancer Institute Newark New Jersey USA; ^2^ Division of Cancer Biology, Department of Radiation Oncology Rutgers New Jersey Medical School Newark New Jersey USA; ^3^ Research Collaboratory for Structural Bioinformatics Protein Data Bank, Institute for Quantitative Biomedicine, Rutgers The State University of New Jersey Piscataway New Jersey USA; ^4^ Department of Chemistry and Chemical Biology Rutgers, The State University of New Jersey Piscataway New Jersey USA; ^5^ Rutgers Artificial Intelligence and Data Science (RAD) Collaboratory Rutgers, The State University of New Jersey Piscataway New Jersey USA; ^6^ Rutgers Cancer Institute New Brunswick New Jersey USA; ^7^ Research Collaboratory for Structural Bioinformatics Protein Data Bank, San Diego Supercomputer Center University of California San Diego La Jolla California USA; ^8^ Division of Hematology/Oncology, Department of Medicine Rutgers New Jersey Medical School Newark New Jersey USA

**Keywords:** autoantibody fingerprinting, cancer targeting, epitope mapping, matrix metalloproteinase 14, phage display

## Abstract

Circulating antibodies in patients with cancer can facilitate the identification of accessible epitopes on autoantigens expressed by tumors. To identify previously unrecognized protein targets in ovarian cancer, we computationally assessed a heptapeptide consensus motif (VPELGHE, flanked by two cysteine residues yielding a cyclic nonapeptide under oxidizing conditions) previously discovered via phage display‐based epitope mapping of autoantibodies in patients. Eight proteins associated with ovarian cancer encompass amino acid sequences similar to the consensus motif and were, therefore, considered as candidate native autoantigens. Among these candidate targets, however, matrix metalloproteinase 14 (*MMP14*) demonstrates gene expression that is both high and negatively correlated with survival in ovarian cancer patient cohorts. MMP14 protein levels are also stable in tumor versus non‐tumor tissues. Moreover, the corresponding heptapeptide mimic in MMP14 occurs within an α‐helical secondary structural element observed in its catalytic domain. These findings demonstrate that a subset of patient‐derived autoantibodies may interact with a previously unknown antigenic epitope found in MMP14 and other MMPs, thereby providing opportunities for the development of new targeted agents.

## Graphical abstract

Circulating autoantibodies are capable of recognizing accessible targets in tumors. In 2004, we previously identified a prominent mimotope (VPELGHE) of autoantibodies in ovarian cancer patients with phage display‐based “fingerprinting.” With the benefit of more than two decades of biomedical literature and open‐access data, we have re‐explored this mimotope with a computational workflow and defined a previously unrecognized region of matrix metalloproteinase 14 (MMP14) and other MMPs as a promising epitope for theranostic applications in ovarian cancer and potentially other malignancies.

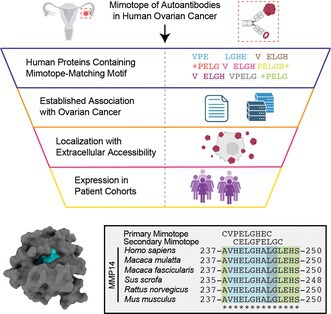

## 1 Introduction

Epitope mapping of autoantibodies from cancer patients using phage display (“fingerprinting”) is a promising approach to identify systemically accessible targets [1, 2, 3]. In 2004, we showed that autoantibodies from an index patient with stage IV ovarian cancer collectively recognize a cyclic nonapeptide, CVPELGHEC, in 86% of recovered sequences after serial selection of a phage display library in immunoglobulins purified from tumor‐derived ascites [3]. At that time, our group used peptide‐based affinity chromatography as a biochemical strategy to putatively isolate, identify, and validate an affinity receptor for these autoantibodies: heat‐shock protein 90 (HSP90) [3]. Over 20 years later, we have revisited the CVPELGHEC motif in the context of large‐scale genomic–proteomic datasets available today. Given that our original experimental strategy for receptor isolation (i) utilized an immortalized human ovarian cancer cell line and (ii) centered on the most prominent protein band immunodetected by the collective mimotope‐binding autoantibodies, we reasoned that in silico epitope mapping and downstream analysis may uncover additional candidate receptors and facilitate advances toward identifying novel antigenic targets.

## 2 Results

**FIGURE 1:**
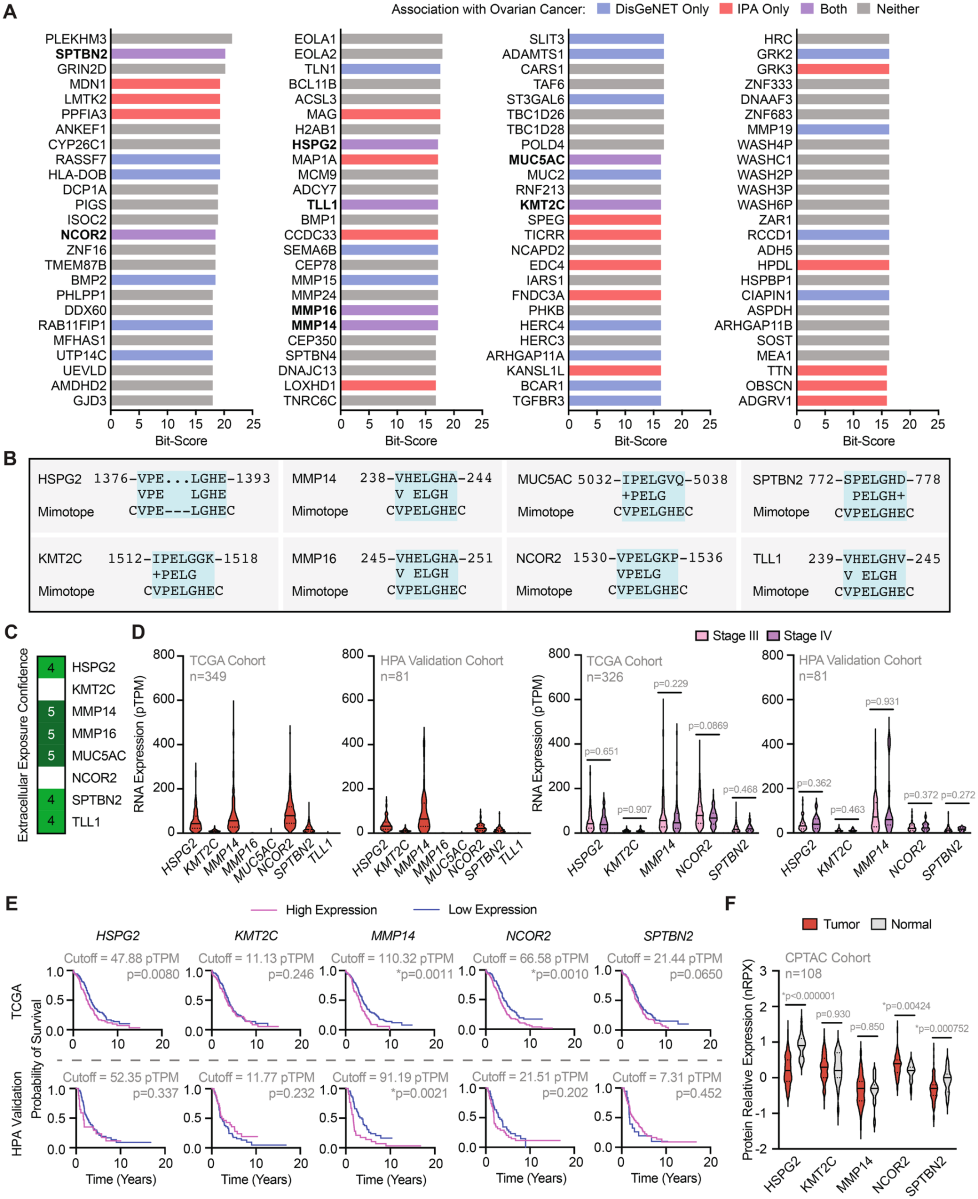
In silico identification of MMP14 as a candidate target for autoantibodies in human ovarian cancer based on the selected peptide mimotope, CVPELGHEC. (A) Top‐scoring proteins in the human proteome (as determined by BLASTP bit‐scores) that map to the consensus motif (VPELGHE). Eight proteins (purple) are associated with ovarian cancer according to both DisGeNET and IPA. (B) Amino acid residue sequence alignments of the mimotope with the eight identified proteins. (C) Confidence of extracellular exposure according to localization as reported by COMPARTMENT (i.e., the knowledge channel) (maximum score = 5). (D) RNA expression represented by pseudo‐transcripts per million (pTPM) of the eight proteins in TCGA and HPA validation human ovarian cancer samples, including stage III versus IV comparisons. Solid line represents median and dotted lines represent quartiles. Data analyzed with Mann–Whitney tests; uncorrected *p* values are listed with Bonferroni‐corrected significance threshold (*α* = 0.05/10 = 0.005). (E) Survival analysis for high versus low expression in TCGA human ovarian cancer samples. Data analyzed with log‐rank (Mantel‐Cox) tests; uncorrected *p* values are listed with Bonferroni‐corrected significance threshold (*α* = 0.05/10 = 0.005). (F) Relative protein quantification represented by normalized relative protein expression (nRPX) of the five expressed proteins in CPTAC human ovarian tumor (*n* = 85) and non‐tumor samples (*n* = 23). Data analyzed with Mann–Whitney tests; uncorrected *p* values are listed with Bonferroni‐corrected significance threshold (*α* = 0.05/5 = 0.01).

The basic local alignment search tool (BLAST) facilitated re‐analysis of the selected consensus motif (VPELGHE) by identifying multiple hits from the human proteome (Figure 1A). The 100 top‐scoring matches were assessed for known associations with ovarian cancer by using (i) DisGeNET primarily for automated scientific literature search (*n* = 28) and (ii) Ingenuity Pathway Analysis (IPA) for stringent evaluation of various data sources (*n* = 25), mutually identifying eight hits (Figure 1B) [4, 5, 6]. Two are not known to have extracellular exposure (Figure 1C), which may vary in a malignant disease context.

We next evaluated RNA expression levels of the eight proteins in The Cancer Genome Atlas (TCGA) and Human Protein Atlas (HPA) validation cohorts of patients with ovarian serous cystadenocarcinoma [7, 8]. *MMP14* and *NCOR2* are most highly expressed in both cohorts (Figure 1D, left), while *MMP16*, *MUC5AC*, and *TLL1* are minimally expressed. Although no differences in RNA expression levels are present in stage III versus IV patients across *HSPG2*, *KMT2C*, *MMP14*, *NCOR2*, and *SPTBN2* (Figure 1D, right), a reduced likelihood of survival is associated with high versus low *MMP14* expression in both cohorts (Figure 1E). Finally, we evaluated relative levels of these proteins in a Clinical Proteomic Tumor Analysis Consortium (CPTAC) cohort [9, 10]; NCOR2 is detected to a higher degree, and HSPG2 and SPTBN2 are detected to a lower degree in ovarian serous cystadenocarcinoma compared to normal tissue (no difference for KMT2C and MMP14) (Figure 1F). Altogether, these findings indicate that MMP14, a zinc (Zn^2+^)‐dependent endopeptidase, and NCOR2, a transcriptional corepressor, are additional promising candidate targets of the autoantibodies from our original study and may become accessible to circulating ligands in ovarian cancer.

**FIGURE 2:**
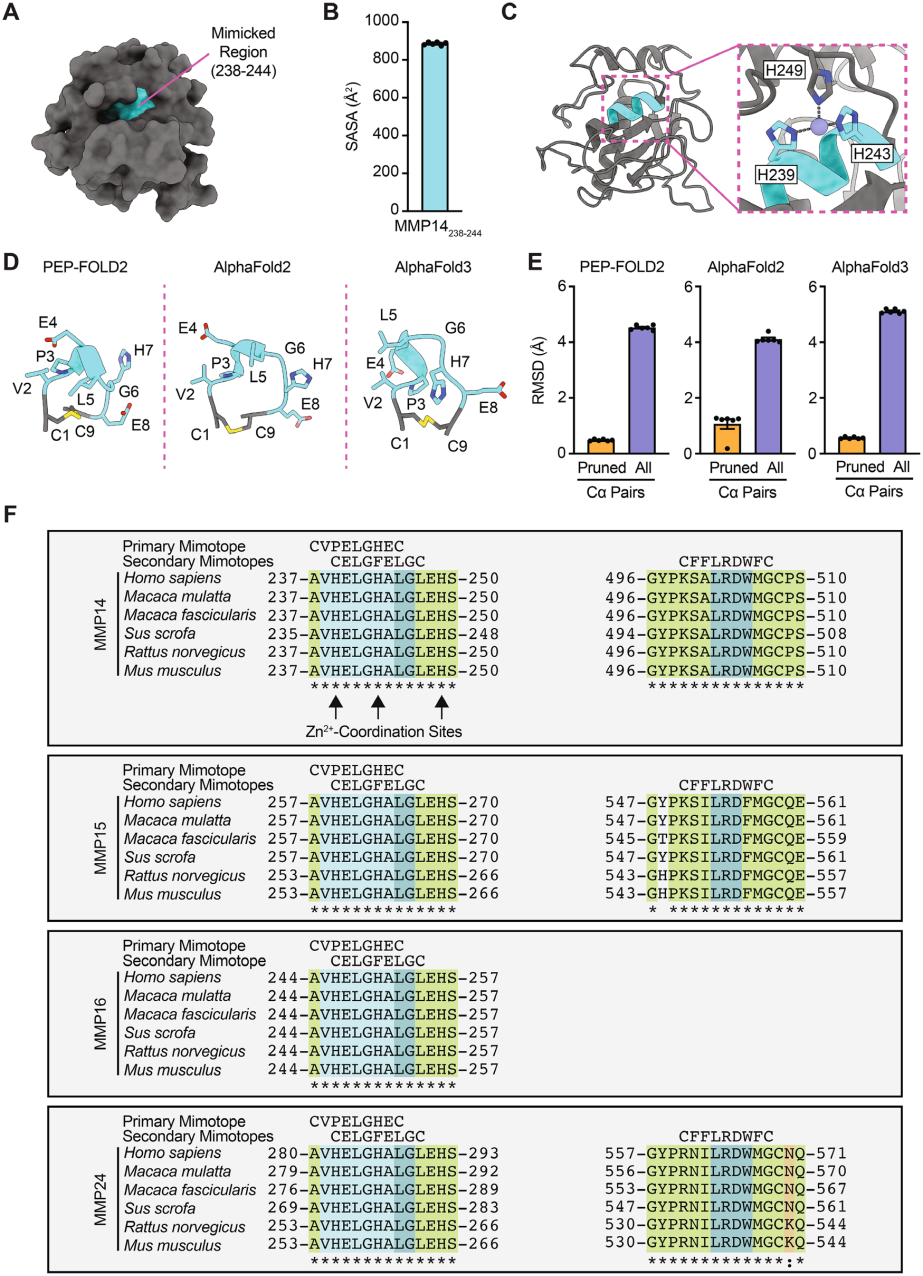
Structural comparison of the proposed native MMP14‐derived VHELGHA epitope (cyan) versus the cyclic nonapeptide, CVPELGHEC. (A) Space‐filling representation of the 3D atomic‐level structure of MMP14 (native VHELGHA in cyan) (PDB ID: 1BUV, chain M). (B) All‐atom solvent‐accessible surface area (SASA) of native VHELGHA across all available region‐containing MMP14 chains in the PDB (PDB IDs: 1BQQ, chain M; 1BUV, chain M; 3MA2, chains A and D; 5H0U, chain A) (*n* = 5) and AlphaFold Protein Structure Database (AF‐P50281‐F1‐model‐v4) (*n* = 1). Data presented as mean ± SEM. (C) Ribbon/stick‐figure representation of MMP14, with inset showing the catalytic center—three histidine residues, two of which occur within the cyan MMP14‐derived proposed epitope—are responsible for binding Zn^2+^ (gray sphere with dashed lines to coordinating N atoms). (D) Predicted 3D atomic‐level structures of the cyclic nonapeptide, CVPELGHEC, by PEP‐FOLD2, AlphaFold2, and AlphaFold3, with the core heptapeptide motif shown in cyan. (E) RMSD of the predicted cyclic nonapeptide structure overlaid with native VHELGHA of MMP14 across all available MMP14 chains. RMSD was calculated for pruned Cα pairs (five for PEP‐FOLD2‐and AlphaFold3‐predicted peptides; four for AlphaFold2‐predicted peptide except three for PDB ID 5H0U, chain A) and all seven Cα pairs. Data presented as mean ± SEM. (F) Multiple sequence alignment of the proposed native VHELGHA epitope (cyan) and adjacent regions of MMP14 and other MMPs based on top BLAST hits of VPELGHE (Figure 1A) from the proteomes of human, rhesus monkey, cynomolgus monkey, pig, rat, and mouse. Secondary mimotopes of autoantibodies identified by Vidal et al. [3] are also aligned.

Visual inspection of three‐dimensional (3D) structures of MMP14 revealed the solvent‐accessible heptapeptide mimotope (VHELGHA, residues 238–244) with five of seven amino acid residues identical to the consensus motif (Figure 2A,B). Remarkably, residues H239 and H243 of MMP14 serve as two of three amino acids responsible for coordinating the Zn^2+^ found within its enzymatic catalytic domain (Figure 2C) [11]. Moreover, an endogenous metalloproteinase inhibitor blocks MMP14 enzymatic function by occupying the open fourth coordination site of Zn^2+^ [12]. Predicted structures of the cyclic nonapeptide, CVPELGHEC, notably contain an α‐helical feature similar to that observed in MMP14 (Figure 2D,E and Figure S1), thereby providing a basis for possible autoantibody recognition of an antigenic epitope. Conversely, the corresponding region (residues 1530–1536) of NCOR2 has not been experimentally resolved and is predicted to be disordered, rendering it a less likely target. Comparative sequence analysis of the mimicked region in MMP14 and other MMPs (i.e., MMP15, MMP16, and MMP24) demonstrates conservation across organisms (Figure 2F), supporting its evolutionary relevance. Finally, secondary mimotopes identified in our original study [3] strikingly mimic an overlapping region (residues 240–246) in addition to a separate, conserved region (residues 500–506) of MMP14 with similar findings in other MMPs.

## 3 Discussion

Computational analysis of a previously identified antigenic mimotope recognized by fingerprinting human autoantibodies from ovarian cancer patients [3] with more than two decades of accumulated biomedical data supports MMP14 and other MMPs as potential targets for discovery and development of translational theranostics. MMP14, which plays a key functional role in extracellular matrix breakdown, is known to be mechanistically associated with epithelial ovarian cancer [13]. We had previously identified and validated HSP90 as one antigen for patient autoantibodies through a stringent biochemical approach; this does not, however, rule out the possibility that HSP90 and MMP14, among other MMPs, may all represent accessible anti‐cancer drug targets. Furthermore, these proteins may be members of the same supramolecular complex since HSP90 functionally interacts with an MMP during tumor angiogenesis [14].

Notwithstanding the advances made by this conceptual study, certain limitations must be acknowledged and addressed. While HSP90 was well‐validated as one target in our original study [3], we are unable to perform similar experiments for the MMPs given the lack of access to the original patient autoantibodies isolated over 20 years ago. Furthermore, the cyclic nonapeptide, CVPELGHEC, had been recognized by ascitic antibodies from patients with stage IV ovarian cancer to a greater degree than stage III in our original study [3], while no difference in gene expression of *MMP14* was identified in stage III versus stage IV ovarian cancer in the current study. It is, therefore, plausible that the newly proposed epitope (residues 238–244) of MMP14 in this study would be targetable in stage IV ovarian cancer due to an acquired conformation with increased accessibility of the identified epitope and/or greater levels of the protein at the membrane or in the extracellular space. Given these open questions, additional studies will be required to elucidate the relationship, if any, between HSP90 and MMP14 in the context of ovarian cancer.

In line with our reasoning and findings, Mazor et al. identified MMP14 as the key antigen for tumor‐reactive autoantibodies in high‐grade serous ovarian carcinoma, consistent with earlier observations that B‐cell involvement in tumors is associated with favorable patient outcomes [15, 16]. Considering that their functional epitope (residues 127–140) maps to a distinct region of MMP14 [15], our previous findings [3] and this post hoc analysis establish the candidacy of a second antigenic epitope in MMP14 and other MMPs for exploration in future studies of human ovarian cancer, and perhaps other malignant solid tumors.

## 4 Methods

### 4.1 Protein–Protein BLAST and Associations with Ovarian Cancer

The consensus motif (VPELGHE) was input into Standard Protein BLAST (National Library of Medicine) and searched against the UniProtKB/Swiss‐Prot (swissprot) database for *Homo sapiens* (Dataset S1 and Supplementary Methods). To assess for associations with ovarian cancer, the 100 top‐scoring proteins according to bit‐score (normalized alignment score generated by BLAST) were input into DisGeNET version 25.1.1 (Table S1) [17] and IPA version 134816949 (QIAGEN) (Table S2) [18]. Additional details are provided in the Supplementary Methods. Subcellular locations were identified with COMPARTMENTS (https://compartments.jensenlab.org/) (Table S3) [19].

### 4.2 Gene Expression, Survival, and Relative Protein Quantification Analysis

To assess gene expression, TCGA (Table S4) [7] and HPA validation (Table S5) [8] datasets of RNA levels in human ovarian serous cystadenocarcinoma samples were retrieved from HPA. For survival analysis, the best‐expression cutoff value for high versus low expression of each gene in each dataset was established by HPA. To assess relative protein quantification, the CPTAC dataset (PDC000110) corresponding to human ovarian serous cystadenocarcinoma and non‐tumor tissue (fallopian tube) [9, 10], intended for single‐protein comparison across samples, was retrieved from HPA (Table S6). HPA version 24.0 was used (https://v24.proteinatlas.org) [20].

### 4.3 Atomic‐Level Structural Visualization and Analysis

Experimental structures of MMP14 originate from the Protein Data Bank [11, 12, 21, 22], and predicted structures of MMP14 and NCOR2 originate from the AlphaFold Protein Structure Database [23, 24]. Structures of cyclic nonapeptide, CVPELGHEC, were predicted with PEP‐FOLD2 [25], AlphaFold2 [23, 26], and AlphaFold3 [27]. Visualization and analysis were performed with UCSF ChimeraX versions 1.7 and 1.8 [28]. Additional details are provided in the Supplementary Methods and Tables S7 and S8.

### 4.4 Statistical Analysis

Data were graphed and analyzed with GraphPad Prism 10 as indicated in the figure legends.

## Author Contributions

C.M., W.A., and R.P. designed the research. C.M. and A.M.I. performed the research. C.M., A.M.I., S.K.B., W.A., and R.P. analyzed the data. C.M., A.M.I., S.K.B., W.A., and R.P. wrote the paper.

## Conflicts of Interest

A.M.I. is a founder and partner of North Horizon, which is engaged in the development of artificial intelligence‐based software. W.A. and R.P. are founders and equity shareholders of PhageNova Bio. R.P. is the Chief Scientific Officer and a paid consultant for PhageNova Bio. W.A. and R.P. are founders and equity shareholders of MBrace Therapeutics; W.A. and R.P. serve as paid consultants for MBrace Therapeutics. W.A. and R.P. have Sponsored Research Agreements (SRAs) in place with PhageNova Bio, MBrace Therapeutics, and Alnylam Pharmaceuticals. These arrangements are managed in accordance with the established institutional conflict‐of‐interest policies of Rutgers, The State University of New Jersey. This study falls outside the scope of these SRAs. C.M. and S.K.B. declare no competing interests.

## Supporting information


**Data S1:** fsb271066‐sup‐0001‐Supinfo.zip.

## Data Availability

All supporting data have been provided in the Results and Supporting Information.
