# Supplementary material for: Matrix Metalloproteinases as Candidate Antigenic Determinants for Anti‐Tumor Autoantibodies in Human Ovarian Cancer: A Post Hoc Analysis
Source: FASEB J. 2025 Sep 23;39(18):e71066. doi: 10.1096/fj.202500739RR (PMC12455903; doi:10.1096/fj.202500739RR)
Supplement: Supplementary file 1 — Data S1: fsb271066‐sup‐0001‐Supinfo.zip. [file FSB2-39-e71066-s001.zip › fsb271066-sup-0001-Supinfo.pdf]

Matrix Metalloproteinases as Candidate Antigenic Determinants for Anti-Tumor Autoantibodies in Human Ovarian Cancer: A *Post Hoc* Analysis

Christopher Markosian, Alexander M. Ille, Stephen K. Burley, Wadih Arap, and Renata Pasqualini

Correspondence should be addressed to Wadih Arap (wadih.arap@rutgers.edu) and/or Renata Pasqualini (renata.pasqualini@rutgers.edu).

Supporting Information Contents

- Supplementary Methods
- Supplementary Figure (S1)
- Supplementary Tables (S1-S8)
- Supplementary Dataset (S1) (separate file)
- Supplementary References

Supplementary Methods

Protein-Protein Basic Local Alignment Search Tool (BLAST) Parameters

Protein-protein BLAST parameters were defined as follows: (i) max target sequences = 100, (ii) expect threshold = 200,000, (iii) word size = 2, (iv) matrix = PAM30, (v) gap costs = existence: 9, extension: 1, and (vi) compositional adjustments = no adjustment. The highest generated bit-score per human protein is presented in **Figure 1A**.

DisGeNET and Ingenuity Pathway Analysis (IPA) Parameters

For DisGeNET, all but six proteins (ASPDH, EOLA2, TBC1D28, TTN, WASH3P, WASH4P) were found. Each protein with associated disease(s) related to ovarian cancer was considered to have a positive relation (**Table S1**). Of note, SPEG was found to have one association (*i.e.*, with “mucinous adenocarcinoma of ovary”), although the supporting information was not provided by the source database and hence not considered positive.

For IPA, all 100 proteins were found. The Path Explorer tool served to identify shortest-pathway connections between “ovarian cancer” as a disease/function and each protein (**Table S2**). IPA parameters were defined as follows (1): (i) direct and indirect interactions, (ii) information limited to the Ingenuity Knowledge Base, (iii) all data sources (*i.e.*, Ingenuity Expert Information, which includes Ingenuity Expert Findings and Ingenuity ExpertAssist Findings, and Ingenuity Supported Third Party Information), (iv) only experimentally observed as the confidence level, (v) multiple species (human, mouse, rat, and uncategorized), (vi) all tissues and cell lines, (vii) all mutations, (viii) all relationship types, (ix) complete publication date range, (x) all node types, (xi) all diseases, and (xii) all biofluids.

Atomic-Level Structural Visualization and Analysis

All-atom solvent-accessible surface area (SASA) was calculated with UCSF ChimeraX (2) by using the "sasa" command for the native VPELGHE-matching region of MMP14 (VHELGHA, residues 238-244) in all applicable PDB chains, including 1BQQ (chain M) (3), 1BUV (chain M) (3), 3MA2 (chains A and D) (4), and 5H0U (chain A) (5), plus the corresponding full-length AlphaFold Protein Structure Database structure (AF-P50281-F1-model-v4) (6, 7) as listed by UniProt (8) (**Table S7**). The conformation of cyclic nonapeptide, CVPELGHEC, was predicted with PEP-FOLD2 (9), AlphaFold2 (6), and AlphaFold3 (10). For PEP-FOLD2, the peptide sequence was input with default settings and a pre-specified disulfide bridge between the flanking cysteine residues (https://mobyle2.rpbs.univ-paris-diderot.fr/cgi-bin/portal.py#forms::PEP-FOLD). The top-scoring model containing a disulfide bridge from a set of 100- and 200-run simulations according to sOPEP energy was selected. For AlphaFold2, the peptide sequence was input into ColabFold with AMBER relaxation enabled (11). Default parameters were used, as follows: (i) template_mode = none, (ii) msa_mode = mmseqs2_uniref_env, (iii) pair_mode = unpaired_paired, (iv) model_type = auto, (v) num_recycles = 3, (vi) recycle_early_stop_tolerance = auto, (vii) relax_max_iterations = 200, (viii) pairing_strategy = greedy, (ix) max_msa = auto, and (x) num_seeds = 1. The top-scoring model, according to per-residue measure of local confidence (pLDDT), was selected. For AlphaFold3, the peptide sequence was input into AlphaFold Server with default settings (https://alphafoldserver.com). The top-scoring model was selected. Root-mean-square deviation (RMSD) values for Cα pairs were calculated by using the Matchmaker function with default settings in UCSF ChimeraX (2). To compare predicted CVPELGHEC structures (PEP-FOLD2, AlphaFold2, and AlphaFold3) *versus* each other, Cα pairs of all nine amino acid residues were overlaid (**Figure S1A**). To compare predicted CVPELGHEC structures *versus* the corresponding mimicked region of MMP14 (VHELGHA, residues 238-244), the seven Cα atoms of VPELGHE (within the nonapeptide) were overlaid with the seven Cα atoms of VHELGHA (within MMP14) (**Figure S1B** and **Table S8**).

Of note, the peptide-matching region (residues 1530-1536) of human NCOR2 (not visualized in this study) is predicted to be disordered according to UniProt (8) and its structure in the AlphaFold Protein Structure Database (AF-Q9Y618-F1-model-v4) (6, 7). Moreover, PEP-FOLD3 (latest version 3.5) was not utilized in this study due to the host notation (https://bioserv.rpbs.univ-paris-diderot.fr/services/PEP-FOLD3/) and recommendation by Lamiable *et al*. that PEP-FOLD2 be utilized for peptides containing disulfide bridges (12). The flanking cysteine residues of CVPELGHEC form a disulfide bridge under oxidizing conditions as investigated by Vidal *et al*. (13).

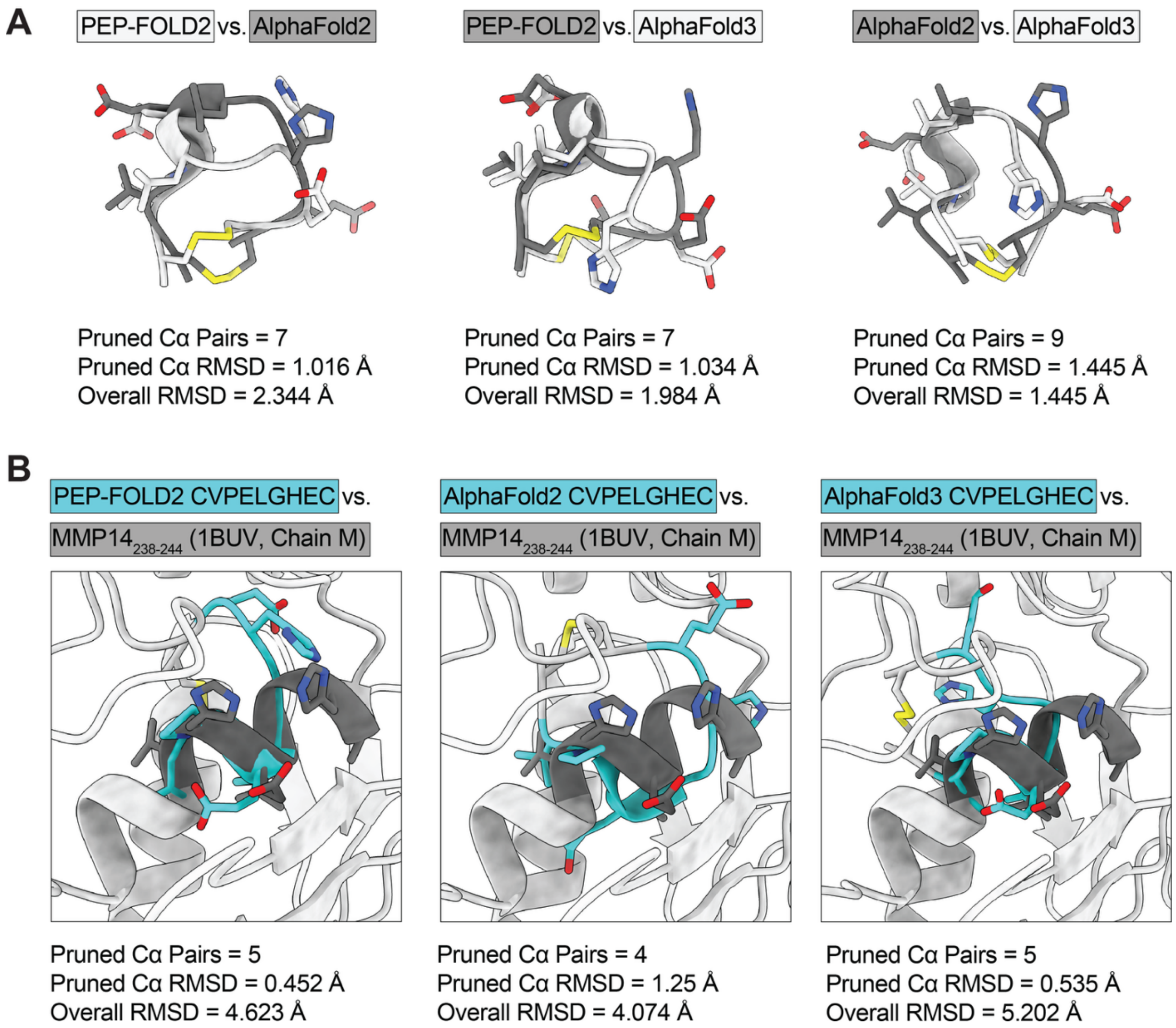

Figure S1: Structural comparisons for predicted peptide structures **(A)** with each other (*i.e.*, nine Cα pairs) and **(B)** with a representative experimental structure of human MMP14 (residues 238-244) (1BUV, chain M) (*i.e.*, seven Cα pairs).

Table S1: Proteins from the top 100 identified with protein-protein BLAST of the mimotope (VPELGHE) (based on bit-score) with at least one connection to a disease encompassed by ovarian cancer according to DisGeNET (n=28).

Gene/Protein	Disease	$Score_{GDA}$	Association Type	Reference
ADAMTS1	malignant neoplasm of ovary	0.2	genetic variation	(14)
	ovarian neoplasm	0.2	genetic variation	(14)
	carcinoma, ovarian epithelial	0.2	genetic variation	(14)
	ovarian carcinoma	0.2	genetic variation	(14)
	ovarian neoplasm	0.2	altered expression	(15)
	malignant neoplasm of ovary	0.2	altered expression	(15)
	ovarian carcinoma	0.2	genetic variation	(16)
	malignant neoplasm of ovary	0.2	genetic variation	(16)
	carcinoma, ovarian epithelial	0.2	genetic variation	(16)
	ovarian neoplasm	0.2	genetic variation	(16)
ARHGAP11A	malignant neoplasm of ovary	0.1	causal or contributing	(17)
	ovarian neoplasm	0.2	causal or contributing	(17)
BCAR1	ovarian serous tumor	0.1	altered expression	(18)
	malignant neoplasm of ovary	0.25	causal or contributing	(19)
	ovarian neoplasm	0.25	causal or contributing	(19)
	carcinoma, ovarian epithelial	0.2	altered expression	(19)
	ovarian carcinoma	0.2	altered expression	(19)
	malignant neoplasm of ovary	0.25	causal or contributing	(20)
	ovarian neoplasm	0.25	altered expression	(20)
	ovarian carcinoma	0.2	causal or contributing	(20)
	carcinoma, ovarian epithelial	0.2	causal or contributing	(20)
	malignant neoplasm of ovary	0.25	causal or contributing	(21)
	ovarian neoplasm	0.25	causal or contributing	(21)
	hereditary breast and ovarian cancer syndrome	0.4	genetic variation	N/A
BMP2	ovarian neoplasm	0.2	altered expression	(22)
	malignant neoplasm of ovary	0.2	altered expression	(22)
	epithelial ovarian cancer	0.1	altered expression	(22)
	carcinoma, ovarian epithelial	0.1	altered expression	(22)
	ovarian neoplasm	0.2	genetic variation	(23)
	malignant neoplasm of ovary	0.2	genetic variation	(23)
	malignant ovarian granulosa cell tumor	0.1	altered expression	(23)
	ovarian granulosa cell tumor	0.1	genetic variation	(23)
	malignant neoplasm of ovary	0.2	causal or contributing	(24)
	ovarian neoplasm	0.2	causal or contributing	(24)
	malignant neoplasm of ovary	0.2	altered expression	(25)
	ovarian neoplasm	0.2	altered expression	(25)
	malignant neoplasm of ovary	0.2	causal or contributing	(26)
	ovarian neoplasm	0.2	causal or contributing	(26)
CIAPIN1	carcinoma, ovarian epithelial	0.1	causal or contributing	(27)
	epithelial ovarian cancer	0.1	causal or contributing	(27)
	ovarian serous adenocarcinoma	0.1	causal or contributing	(28)
GRK2	ovarian neoplasm	0.2	altered expression	(29)
	epithelial ovarian cancer	0.2	altered expression	(29)
	epithelial tumor of ovary	0.2	altered expression	(29)
	carcinoma, ovarian epithelial	0.2	altered expression	(29)
	malignant neoplasm of ovary	0.2	altered expression	(29)
	malignant neoplasm of ovary	0.2	causal or contributing	(30)
	ovarian neoplasm	0.2	causal or contributing	(30)

	epithelial tumor of ovary	0.2	altered expression	(31)
	carcinoma, ovarian epithelial	0.2	altered expression	(31)
	epithelial ovarian cancer	0.2	altered expression	(31)
	malignant neoplasm of ovary	0.2	altered expression	(32)
	ovarian neoplasm	0.2	altered expression	(32)
	dysgerminoma of ovary	0.1	altered expression	(33)
HERC4	malignant neoplasm of ovary	0.1	altered expression	(34)
	ovarian neoplasm	0.1	altered expression	(34)
HLA-DOB	ovarian neoplasm	0.1	causal or contributing	(35)
	malignant neoplasm of ovary	0.1	causal or contributing	(35)
HSPG2	malignant neoplasm of ovary	0.2	altered expression	(36)
	ovarian neoplasm	0.2	altered expression	(36)
	malignant neoplasm of ovary	0.2	altered expression	(37)
	ovarian neoplasm	0.2	altered expression	(37)
KMT2C	malignant neoplasm of ovary	0.2	genetic variation	(38)
	ovarian neoplasm	0.2	genetic variation	(38)
	malignant neoplasm of ovary	0.2	genetic variation	(39)
	ovarian neoplasm	0.2	genetic variation	(39)
MMP14	ovarian neoplasm	0.35	altered expression	(40)
	malignant neoplasm of ovary	0.35	altered expression	(40)
	carcinoma, ovarian epithelial	0.25	altered expression	(40)
	ovarian carcinoma	0.25	altered expression	(40)
	ovarian neoplasm	0.35	altered expression	(41)
	malignant neoplasm of ovary	0.35	altered expression	(41)
	carcinoma, ovarian epithelial	0.25	causal or contributing	(41)
	ovarian carcinoma	0.25	causal or contributing	(41)
	epithelial tumor of ovary	0.1	altered expression	(41)
	malignant neoplasm of ovary	0.35	altered expression	(42)
	carcinoma, ovarian epithelial	0.25	altered expression	(42)
	ovarian carcinoma	0.25	altered expression	(42)
	malignant neoplasm of ovary	0.35	altered expression	(43)
	carcinoma, ovarian epithelial	0.25	altered expression	(43)
	ovarian carcinoma	0.25	altered expression	(43)
	ovarian neoplasm	0.35	altered expression	(44)
	malignant neoplasm of ovary	0.35	altered expression	(44)
	malignant neoplasm of ovary	0.35	altered expression	(45)
	carcinoma, ovarian epithelial	0.25	altered expression	(45)
	ovarian carcinoma	0.25	altered expression	(45)
	ovarian neoplasm	0.35	altered expression	(46)
	malignant neoplasm of ovary	0.35	altered expression	(46)
	ovarian neoplasm	0.35	causal or contributing	(47)
	malignant neoplasm of ovary	0.35	causal or contributing	(47)
	malignant neoplasm of ovary	0.35	altered expression	(48)
	ovarian neoplasm	0.35	altered expression	(48)
	carcinoma, ovarian epithelial	0.25	causal or contributing	(49)
	ovarian carcinoma	0.25	causal or contributing	(49)
	malignant neoplasm of ovary	0.35	causal or contributing	(49)
	ovarian neoplasm	0.35	causal or contributing	(50)
	malignant neoplasm of ovary	0.35	causal or contributing	(50)
	malignant neoplasm of ovary	0.35	altered expression	(51)
	carcinoma, ovarian epithelial	0.25	altered expression	(51)
	stage IV ovarian carcinoma	0.1	causal or contributing	(51)
	ovarian carcinoma	0.25	altered expression	(51)
	borderline ovarian tumour	0.1	altered expression	(51)
	malignant neoplasm of ovary	0.35	altered expression	(52)
	ovarian neoplasm	0.35	altered expression	(52)
	malignant neoplasm of ovary	0.35	altered expression	(53)

	ovarian neoplasm	0.35	altered expression	(53)
	carcinoma, ovarian epithelial	0.25	altered expression	(54)
	epithelial ovarian cancer	0.2	altered expression	(54)
	ovarian neoplasm	0.35	causal or contributing	(55)
	malignant neoplasm of ovary	0.35	causal or contributing	(55)
	carcinoma, ovarian epithelial	0.25	altered expression	(56)
	epithelial ovarian cancer	0.2	altered expression	(56)
	ovarian neoplasm	0.35	causal or contributing	(57)
	malignant neoplasm of ovary	0.35	causal or contributing	(57)
	ovarian neoplasm	0.35	causal or contributing	(58)
	malignant neoplasm of ovary	0.35	causal or contributing	(58)
	ovarian neoplasm	0.35	causal or contributing	(59)
	malignant neoplasm of ovary	0.35	causal or contributing	(59)
	ovarian neoplasm	0.35	post translational modification	(60)
	malignant neoplasm of ovary	0.35	post translational modification	(60)
	ovarian neoplasm	0.35	causal or contributing	(61)
	ovarian serous tumor	0.1	altered expression	(62)
	ovarian neoplasm	0.35	causal or contributing	(63)
	malignant neoplasm of ovary	0.35	causal or contributing	(63)
	ovarian neoplasm	0.35	genetic variation	(64)
	malignant neoplasm of ovary	0.35	genetic variation	(64)
	ovarian neoplasm	0.35	causal or contributing	(65)
	malignant neoplasm of ovary	0.35	causal or contributing	(65)
	ovarian neoplasm	0.35	causal or contributing	(66)
	malignant neoplasm of ovary	0.35	causal or contributing	(66)
MMP15	ovarian carcinoma	0.1	altered expression	(67)
	ovarian neoplasm	0.1	altered expression	(67)
	malignant neoplasm of ovary	0.1	altered expression	(67)
	carcinoma, ovarian epithelial	0.1	altered expression	(67)
MMP16	ovarian neoplasm	0.1	causal or contributing	(68)
	malignant neoplasm of ovary	0.1	causal or contributing	(68)
	ovarian serous tumor	0.1	altered expression	(69)
MMP19	malignant neoplasm of ovary	0.2	altered expression	(70)
	ovarian neoplasm	0.2	altered expression	(70)
	ovarian neoplasm	0.2	altered expression	(71)
	malignant neoplasm of ovary	0.2	altered expression	(71)
MUC2	malignant neoplasm of ovary	0.2	altered expression	(72)
	carcinoma, ovarian epithelial	0.2	altered expression	(72)
	ovarian carcinoma	0.2	altered expression	(72)
	endometroid carcinoma ovary	0.1	altered expression	(73)
	ovarian carcinoma	0.2	causal or contributing	(73)
	malignant neoplasm of ovary	0.2	causal or contributing	(73)
	ovarian neoplasm	0.2	altered expression	(73)
	carcinoma, ovarian epithelial	0.2	causal or contributing	(73)
	ovarian mucinous tumor	0.2	altered expression	(74)
	ovarian mucinous tumor	0.2	altered expression	(75)
	carcinoma, ovarian epithelial	0.2	causal or contributing	(76)
	malignant neoplasm of ovary	0.2	causal or contributing	(76)
	ovarian carcinoma	0.2	causal or contributing	(76)
	ovarian neoplasm	0.2	altered expression	(77)
	malignant neoplasm of ovary	0.2	altered expression	(77)
	ovarian mucinous tumor	0.2	altered expression	(78)
	ovarian mucinous tumor	0.2	causal or contributing	(79)
	ovarian mucinous tumor	0.2	altered expression	(80)
MUC5AC	epithelial ovarian cancer	0.1	altered expression	(81)
	carcinoma, ovarian epithelial	0.2	altered expression	(81)
	malignant neoplasm of ovary	0.2	altered expression	(72)

	ovarian carcinoma	0.2	altered expression	(72)
	carcinoma, ovarian epithelial	0.2	altered expression	(72)
	endometrioid carcinoma ovary	0.1	altered expression	(73)
	malignant neoplasm of ovary	0.2	causal or contributing	(73)
	ovarian carcinoma	0.2	causal or contributing	(73)
	carcinoma, ovarian epithelial	0.2	causal or contributing	(73)
	ovarian neoplasm	0.2	altered expression	(73)
	ovarian mucinous tumor	0.2	altered expression	(82)
	mucinous adenocarcinoma of ovary	0.1	causal or contributing	(83)
	ovarian mucinous tumor	0.2	altered expression	(78)
	ovarian serous adenocarcinoma	0.1	causal or contributing	(84)
	ovarian mucinous tumor	0.2	causal or contributing	(79)
	malignant neoplasm of ovary	0.2	altered expression	(85)
	ovarian neoplasm	0.2	altered expression	(85)
	ovarian mucinous tumor	0.2	altered expression	(80)
	malignant neoplasm of ovary	0.2	causal or contributing	(86)
	ovarian neoplasm	0.2	causal or contributing	(86)
	ovarian mucinous tumor	0.2	causal or contributing	(87)
	malignant neoplasm of ovary	0.2	causal or contributing	(88)
	ovarian neoplasm	0.2	causal or contributing	(88)
NCOR2	malignant neoplasm of ovary	0.2	altered expression	(89)
	ovarian neoplasm	0.2	altered expression	(89)
	ovarian neoplasm	0.2	altered expression	(90)
	malignant neoplasm of ovary	0.2	altered expression	(90)
	ovarian neoplasm	0.2	altered expression	(91)
	malignant neoplasm of ovary	0.2	altered expression	(91)
	carcinoma, ovarian epithelial	0.1	altered expression	(91)
	epithelial ovarian cancer	0.1	altered expression	(91)
RAB11FIP1	malignant neoplasm of ovary	0.2	altered expression	(92)
	ovarian neoplasm	0.2	altered expression	(92)
	ovarian neoplasm	0.2	causal or contributing	(93)
	malignant neoplasm of ovary	0.2	causal or contributing	(93)
	malignant neoplasm of ovary	0.2	post translational modification	(94)
	ovarian neoplasm	0.2	post translational modification	(94)
	ovarian neoplasm	0.2	altered expression	(95)
	malignant neoplasm of ovary	0.2	altered expression	(95)
RASSF7	ovarian neoplasm	0.2	genetic variation	(96)
	malignant neoplasm of ovary	0.2	genetic variation	(96)
	ovarian neoplasm	0.2	genetic variation	(97)
	malignant neoplasm of ovary	0.2	genetic variation	(97)
	epithelial ovarian cancer	0.1	genetic variation	(97)
	carcinoma, ovarian epithelial	0.1	genetic variation	(97)
RCCD1	ovarian neoplasm	0.1	genetic variation	(98)
	malignant neoplasm of ovary	0.1	genetic variation	(98)
SEMA6B	ovarian neoplasm	0.1	causal or contributing	(99)
	malignant neoplasm of ovary	0.1	causal or contributing	(99)
SLIT3	neoplasm of uncertain or unknown behavior of ovary	0.05	genetic variation	(100)
SPTBN2	ovarian neoplasm	0.2	causal or contributing	(101)
	malignant neoplasm of ovary	0.2	causal or contributing	(101)
	malignant neoplasm of ovary	0.2	causal or contributing	(102)
	ovarian neoplasm	0.2	causal or contributing	(102)
ST3GAL6	malignant neoplasm of ovary	0.1	altered expression	(103)
TGFBR3	ovarian granulosa cell tumor	0.1	altered expression	(104)
	stromal tumor of ovary	0.1	altered expression	(105)
	ovarian neoplasm	0.25	altered expression	(106)
	malignant neoplasm of ovary	0.25	altered expression	(106)

	malignant ovarian granulosa cell tumor	0.1	causal or contributing	(107)
	ovarian neoplasm	0.25	altered expression	(108)
	malignant neoplasm of ovary	0.25	altered expression	(108)
	ovarian neoplasm	0.25	causal or contributing	(109)
	malignant neoplasm of ovary	0.25	causal or contributing	(109)
TLL1	malignant neoplasm of ovary	0.1	causal or contributing	(110)
	ovarian neoplasm	0.1	causal or contributing	(110)
	carcinoma, ovarian epithelial	0.1	causal or contributing	(111)
	epithelial ovarian cancer	0.1	causal or contributing	(111)
TLN1	carcinoma, ovarian epithelial	0.1	altered expression	(112)
	epithelial ovarian cancer	0.1	altered expression	(112)
	ovarian serous tumor	0.1	causal or contributing	(113)
	ovarian serous adenocarcinoma	0.1	altered expression	(114)
UTP14C	ovarian neoplasm	0.2	altered expression	(115)
	malignant neoplasm of ovary	0.2	altered expression	(115)
	ovarian neoplasm	0.2	altered expression	(116)
	malignant neoplasm of ovary	0.2	altered expression	(116)

Note: Genes/proteins with at least one connection to a disease within ovarian cancer according to DisGeNET version 25.1.1 as of 1 May 2025 are listed. Disease terms include: borderline ovarian tumour; carcinoma, ovarian epithelial; dysgerminoma of ovary; endometrioid carcinoma ovary; epithelial ovarian cancer; hereditary breast and ovarian cancer syndrome; malignant neoplasm of ovary; malignant ovarian granulosa cell tumor; mucinous adenocarcinoma of ovary; neoplasm of uncertain or unknown behavior of ovary; ovarian carcinoma; ovarian granulosa cell tumor; ovarian mucinous tumor; ovarian neoplasm; ovarian serous adenocarcinoma; ovarian serous tumor; stage IV ovarian carcinoma; and stromal tumor of ovary.

Table S2: Proteins from the top 100 identified with protein-protein BLAST of the mimotope (VPELGHE) (based on bit-score) with at least one connection to "ovarian cancer" according to Ingenuity Pathway Analysis (IPA) (n=25).

Gene/Protein	Connection to Ovarian Cancer	Source	Reference
ADGRV1	*Mutant human ADGRV1 gene (c.7733G>A translating to p.G2578E [somatic missense]) is observed with adenosquamous carcinoma in human ovary (2/75 samples).*	COSMIC	-
	Mutant human ADGRV1 gene (SNP substitution, allelic variations: G/? (rs58142303)) is associated with ovarian carcinoma in human (P = 7.0E-6).	An Open Access Database of Genome-Wide Association Results	(117)
CCDC33	*Mutant human CCDC33 gene (c.1938+468G>C [somatic]) is observed with adenosquamous carcinoma in human ovary (2/17 samples).*	COSMIC	-
EDC4	*Mutant human EDC4 gene (c.1700C>G translating to p.P567R [somatic missense]) is observed with adenosquamous carcinoma in human ovary (COSMIC: observed in 3 of 7 samples).<br>Mutant human EDC4 gene (c.2765A>G translating to p.K922R [somatic missense]) is observed with adenosquamous carcinoma in human ovary (COSMIC: observed in 3 of 7 samples).*	COSMIC	-
FNDC3A	*Mutant human FNDC3A gene (c.1463A>C translating to p.N488T [somatic missense]) is observed with serous adenocarcinoma in human ovary (COSMIC: observed in 4 of 4 samples).*	COSMIC	(118)
GRK3	*Mutant human GRK3 gene (g.25722290G>T [somatic]) is observed with serous adenocarcinoma in human ovary (COSMIC: observed in 2 of 6 samples).*	COSMIC	(119)
HPDL	*Mutant human HPDL gene (c.763G>T translating to p.G255C [somatic missense]) is observed with serous adenocarcinoma in human ovary (COSMIC: observed in 2 of 2 samples).*	COSMIC	(118)
HSPG2	*Mutant human HSPG2 gene (c.10029G>A) is observed with mucinous adenocarcinoma in human ovary (COSMIC: observed in 2 of 153 samples).<br>Mutant human HSPG2 gene (c.10245C>T) is observed with mucinous adenocarcinoma in human ovary (COSMIC: observed in 2 of 153 samples).*	COSMIC	(120)
KANSL1L	*Mutant human KANSL1L gene (c.744C>G [somatic]) is observed with serous adenocarcinoma in human ovary (COSMIC: observed in 4 of 4 samples).*	COSMIC	(118)
KMT2C	*Mutant human MLL3 [KMT2C] gene (unspecified DNA mutation) is associated with ovarian serous carcinoma in human.*	Ingenuity Expert Findings	(121)
	Mutant human KMT2C gene (c.5150A>G translating to p.K1717R [missense]) is observed with endometrioid carcinoma in human ovary (COSMIC: observed in 2 of 22 samples).	COSMIC	(122)
	Mutant human KMT2C gene (c.1081A>G translating to p.T361A [somatic missense]) is observed with serous adenocarcinoma in human ovary (COSMIC: observed in 2 of 3 samples).	COSMIC	(118)
	Mutant human KMT2C gene (c.8252C>T translating to p.S2751L [somatic missense]) is observed with adenosquamous carcinoma in human ovary (COSMIC: observed in 2 of 50 samples).	COSMIC	-
LMTK2	*Mutant human LMTK2 gene (c.3411C>G [somatic]) is observed with adenosquamous carcinoma in human ovary (COSMIC: observed in 2 of 17 samples).*	COSMIC	-
LOXHD1	*Mutant human LOXHD1 gene (c.1093C>A) is observed with mucinous adenocarcinoma in human ovary (COSMIC: observed in 2 of 153 samples).<br>Mutant human LOXHD1 gene (c.1381C>A) is observed with mucinous adenocarcinoma in human ovary (COSMIC: observed in 2 of 153 samples).<br>Mutant human LOXHD1 gene (g.46524734G>T) is observed with mucinous adenocarcinoma in human ovary (COSMIC: observed in 2 of 153 samples).<br>Mutant human LOXHD1 gene (c.127G>A translating to p.E43K [missense]) is observed with mucinous adenocarcinoma in human ovary (COSMIC: observed in 2 of 153 samples).*	COSMIC	(120)
	Mutant human LOXHD1 gene (c.5127C>T [somatic]) is observed with serous adenocarcinoma in human ovary (COSMIC: observed in 4 of 4 samples).	COSMIC	(123)

MAG	Mutant human MAG gene (c.808G>A translating to p.V270M [somatic missense]) is observed with adenosquamous carcinoma in human ovary (COSMIC: observed in 3 of 15 samples).	COSMIC	-
MAP1A	Mutant human MAP1A gene (c.1313T>G translating to p.M438R [somatic missense]) is observed with serous adenocarcinoma in human ovary (COSMIC: observed in 4 of 4 samples).	COSMIC	(118)
MDN1	Mutant human MDN1 gene (c.11830-1G>A [somatic]) is observed with serous adenocarcinoma in human ovary (COSMIC: observed in 3 of 3 samples).	COSMIC	(118)
	Mutant human MDN1 gene (c.2294T>G translating to p.L765R [somatic missense]) is observed with adenosquamous carcinoma in human ovary (COSMIC: observed in 2 of 31 samples).<br>Mutant human MDN1 gene (c.3865G>A translating to p.E1289K [somatic missense]) is observed with adenosquamous carcinoma in human ovary (COSMIC: observed in 2 of 31 samples).	COSMIC	-
MMP14	Upregulation of MT1-MMP [MMP14] protein in metastatic ovarian carcinoma cells from peritoneum is associated with metastatic ovarian cancer in human.	Ingenuity Expert Findings	(50)
MMP16	Mutant human MMP16 gene (c.923dup translating to p.H309Tfs*8 [somatic]) is observed with serous adenocarcinoma in human ovary (COSMIC: observed in 2 of 11 samples).	COSMIC	(119)
MUC5AC	Mutant human MUC5AC gene (c.1840G>A translating to p.E614K [somatic missense]) is observed with adenosquamous carcinoma in human ovary (COSMIC: observed in 2 of 6 samples).	COSMIC	-
NCOR2	Upregulation of human NCOR2 mRNA in tumor is associated with ovarian cancer in Homo sapiens (human).	Ingenuity Expert Findings	(124)
	Mutant human NCOR2 gene (g.124325411G>C [somatic]) is observed with adenosquamous carcinoma in human ovary (COSMIC: observed in 2 of 35 samples).<br>Mutant human NCOR2 gene (g.124344767G>A [somatic]) is observed with adenosquamous carcinoma in human ovary (COSMIC: observed in 2 of 35 samples).<br>Mutant human NCOR2 gene (c.1792dup translating to p.Q598Pfs*13 [somatic]) is observed with serous adenocarcinoma in human ovary (COSMIC: observed in 2 of 15 samples).<br>Mutant human NCOR2 gene (c.4514C>T translating to p.A1505V [somatic missense]) is observed with adenosquamous carcinoma in human ovary (COSMIC: observed in 2 of 35 samples).<br>Mutant human NCOR2 gene (c.4544C>T translating to p.A1515V [somatic missense]) is observed with adenosquamous carcinoma in human ovary (COSMIC: observed in 2 of 35 samples).	COSMIC	-
	Mutant human NCOR2 gene (c.1792dup translating to p.Q598Pfs*13 [somatic]) is observed with serous adenocarcinoma in human ovary (COSMIC: observed in 2 of 14 samples).	COSMIC	(119)
OBSCN	Mutant human OBSCN gene (c.11059G>A translating to p.V3687M [somatic missense]) is observed with carcinoma in human ovary (COSMIC: observed in 6 of 6 samples).<br>Mutant human OBSCN gene (c.12346G>A translating to p.V4116M [somatic missense]) is observed with carcinoma in human ovary (COSMIC: observed in 6 of 6 samples).	COSMIC	(125)
	Mutant human OBSCN gene (c.3041T>C translating to p.M1014T [somatic missense]) is observed with serous adenocarcinoma in human ovary (COSMIC: observed in 2 of 32 samples).<br>Mutant human OBSCN gene (c.3317T>C translating to p.M1106T [somatic missense]) is observed with serous adenocarcinoma in human ovary (COSMIC: observed in 2 of 32 samples).	COSMIC	(119)
	Mutant human OBSCN gene (c.3041T>C translating to p.M1014T [somatic missense]) is observed with serous adenocarcinoma in human ovary (COSMIC: observed in 2 of 49 samples).<br>Mutant human OBSCN gene (c.3317T>C translating to p.M1106T [somatic missense]) is observed with serous adenocarcinoma in human ovary (COSMIC: observed in 2 of 49 samples).<br>Mutant human OBSCN gene (c.19216C>G translating to p.Q6406E [somatic missense]) is observed with adenosquamous carcinoma in human ovary (COSMIC: observed in 2 of 37 samples).	COSMIC	-
PPFIA3	Mutant human PPFIA3 gene (g.49148140C>A [somatic]) is observed with adenosquamous carcinoma in human ovary (COSMIC: observed in 2 of 5 samples).	COSMIC	-
SPEG	Mutant human SPEG gene (c.7599G>C [somatic]) is observed with adenosquamous carcinoma in human ovary (COSMIC: observed in 2 of 17 samples).	COSMIC	-

SPTBN2	*Mutant human SPTBN2 gene (g.66701038C>T [somatic]) is observed with serous adenocarcinoma in human ovary (COSMIC: observed in 4 of 4 samples).*	COSMIC	(118)
TICRR	*Mutant human TICRR gene (c.655-2A>T [somatic]) is observed with serous adenocarcinoma in human ovary (COSMIC: observed in 2 of 12 samples).*	COSMIC	(119)
TLL1	*Mutant human TLL1 gene (c.2029G>T translating to p.E677* [somatic nonsense]) is observed with mucinous adenocarcinoma in human ovary (COSMIC: observed in 2 of 155 samples).*	COSMIC	(120)
TTN	*Mutant human TTN gene (c.61539C>T [somatic]) is observed with carcinoma in human ovary (COSMIC: observed in 3 of 9 samples).*<br>*Mutant human TTN gene (c.81030C>T [somatic]) is observed with carcinoma in human ovary (COSMIC: observed in 3 of 9 samples).*<br>*Mutant human TTN gene (c.13282+32852C>T [somatic]) is observed with carcinoma in human ovary (COSMIC: observed in 6 of 9 samples).*<br>*Mutant human TTN gene (c.25816C>T translating to p.L8606F [somatic missense]) is observed with carcinoma in human ovary (COSMIC: observed in 6 of 9 samples).*	COSMIC	(125)
	Mutant human TTN gene (c.35956T>C [somatic]) is observed with adenosquamous carcinoma in human ovary (COSMIC: observed in 3 of 64 samples).<br>*Mutant human TTN gene (c.55447T>C [somatic]) is observed with adenosquamous carcinoma in human ovary (COSMIC: observed in 3 of 64 samples).*<br>*Mutant human TTN gene (c.30128G>T translating to p.G10043V [somatic missense]) is observed with adenosquamous carcinoma in human ovary (COSMIC: observed in 2 of 64 samples).*<br>*Mutant human TTN gene (c.49619G>T translating to p.G16540V [somatic missense]) is observed with adenosquamous carcinoma in human ovary (COSMIC: observed in 2 of 64 samples).*	COSMIC	-
	Mutant human TTN gene (c.10222+3485G>C [somatic heterozygous]) is observed with carcinoma in human ovary (COSMIC: observed in 2 of 3 samples).	COSMIC	(126)
	Mutant human TTN gene (c.1209C>A translating to p.S403R [somatic missense]) is observed with serous adenocarcinoma in human ovary (COSMIC: observed in 4 of 8 samples).<br>*Mutant human TTN gene (c.28052G>C translating to p.G9351A [somatic missense]) is observed with serous adenocarcinoma in human ovary (COSMIC: observed in 4 of 8 samples).*<br>*Mutant human TTN gene (c.47543G>C translating to p.G15848A [somatic missense]) is observed with serous adenocarcinoma in human ovary (COSMIC: observed in 4 of 8 samples).*<br>*Mutant human TTN gene (c.58826A>G translating to p.K19609R [somatic missense]) is observed with serous adenocarcinoma in human ovary (COSMIC: observed in 4 of 8 samples).*<br>*Mutant human TTN gene (c.78317A>G translating to p.K26106R [somatic missense]) is observed with serous adenocarcinoma in human ovary (COSMIC: observed in 4 of 8 samples).*	COSMIC	(118)
	Mutant human TTN gene (c.24595G>A translating to p.E8199K [somatic missense]) is observed with serous adenocarcinoma in human ovary (COSMIC: observed in 2 of 3 samples).<br>*Mutant human TTN gene (c.44086G>A translating to p.E14696K [somatic missense]) is observed with serous adenocarcinoma in human ovary (COSMIC: observed in 2 of 3 samples).*	COSMIC	(123)
Note: Genes/proteins with at least one connection to a disease within ovarian cancer according to IPA version 134816949 as of 30 April 2025 are listed.			

Table S3: Subcellular localizations of proteins with a connection to ovarian cancer in both DisGeNET and IPA according to the knowledge channel of COMPARTMENT.

Protein	Subcellular Localization	Source	Evidence	Confidence
HSPG2	Plasma membrane protein complex	ARUK-UCL	TAS	4/5
	Basement membrane	ARUK-UCL	TAS	4/5
	Extracellular space	UniProtKB	HDA	4/5
	Golgi lumen	Reactome	TAS	4/5
	Plasma membrane	Reactome	TAS	4/5
	Focal adhesion	UniProtKB	HDA	4/5
	Collagen-containing extracellular matrix	UniProtKB	HDA	4/5
	Extracellular exosome	UniProtKB	HDA	4/5
	Lysosomal lumen	Reactome	TAS	4/5
	Extracellular region	BHF-UCL	HDA	3/5
	Neuron projection	GO_Central	IBA	3/5
KMT2C	Nucleus	MGI	IDA	5/5
	MLL3/4 complex	UniProtKB	IDA	5/5
	Cytosol	HPA	IDA	4/5
	Nucleoplasm	HPA	IDA	4/5
	MLL3/4 complex	InterPro	IEA	2/5
	MLL3/4 complex	InterPro	IEA	2/5
	MLL3/4 complex	InterPro	IEA	2/5
	MLL3/4 complex	InterPro	IEA	2/5
	MLL3/4 complex	InterPro	IEA	2/5
	MLL3/4 complex	InterPro	IEA	2/5
MMP14	Plasma membrane	UniProtKB	IDA	5/5
	Macropinosome	UniProtKB	IDA	5/5
	Cytoplasmic vesicle	UniProtKB	IDA	5/5
	Focal adhesion	UniProtKB	HDA	4/5
	Intermediate filament cytoskeleton	HPA	IDA	4/5
	Golgi lumen	Reactome	TAS	4/5
	Cytosol	HPA	IDA	4/5
	Melanosome	UniProtKB-SubCell	IEA	3/5
	Nucleus	CAFA	IMP	3/5

	Extracellular space	CAFA	IMP	3/5
	Extracellular matrix	InterPro	IEA	2/5
MMP16	Cell surface	UniProtKB	CURATED	5/5
	Golgi lumen	Reactome	TAS	4/5
	Plasma membrane	ParkinsonsUK-UCL	IDA	4/5
	Extracellular space	GO_Central	IBA	3/5
	Extracellular matrix	InterPro	IEA	2/5
	Extracellular matrix	InterPro	IEA	2/5
MUC5AC	Extracellular space	UniProtKB	IDA	5/5
	Mucus layer	MGI	IDA	5/5
	Golgi lumen	Reactome	TAS	4/5
	Plasma membrane	Reactome	TAS	4/5
	Extracellular exosome	UniProtKB	HDA	4/5
	Extracellular space	GO_Central	IBA	3/5
NCOR2	Nucleus	MGI	IDA	5/5
	Nuclear matrix	UniProtKB	IDA	5/5
	Nuclear body	MGI	IDA	5/5
	Nucleoplasm	HPA	IDA	4/5
	Chromatin	BHF-UCL	IDA	4/5
	Nucleoplasm	HPA	IDA	4/5
	Nucleoplasm	HPA	IDA	4/5
	Membrane	UniProtKB	HDA	4/5
	Nucleoplasm	HPA	IDA	4/5
	Nucleoplasm	HPA	IDA	4/5
	Nucleoplasm	HPA	IDA	4/5
SPTBN2	GO:0008091	UniProtKB	IDA	5/5
	Cytoskeleton	UniProtKB	CURATED	5/5
	Cell cortex	UniProtKB	CURATED	5/5
	Plasma membrane	HPA	IDA	4/5
	Plasma membrane	HPA	IDA	4/5
	Intracellular membrane-bounded organelle	HPA	IDA	4/5
	Extracellular space	UniProtKB	HDA	4/5
	Cytosol	HPA	IDA	4/5
	Intracellular membrane-bounded organelle	HPA	IDA	4/5

	Cytosol	HPA	IDA	4/5
	Cortical actin cytoskeleton	GO_Central	IBA	3/5
	Cell junction	GO_Central	IBA	3/5
	Cell projection	GO_Central	IBA	3/5
	Neuronal cell body	Ensembl	IEA	2/5
	Paranodal junction	Ensembl	IEA	2/5
	Apical plasma membrane	Ensembl	IEA	2/5
	Parallel fiber to Purkinje cell synpase	Ensembl	IEA	2/5
	Presynapse	Ensembl	IEA	2/5
	Glutamatergic synapse	Ensembl	IEA	2/5
	Postsynaptic spectrin-associated cytoskeleton	Ensembl	IEA	2/5
TLL1	Extracellular region	Reactome	TAS	4/5
	Extracellular space	GO_Central	IBA	3/5
	Integral component of membrane	UniProtKB-KW	IEA	3/5
	Integral component of membrane	UniProtKB-KW	IEA	3/5
Note: Compartments per protein as of 3 May 2025 are listed.				

Table S4: Data corresponding to human ovarian cancer samples from The Cancer Genome Atlas (TCGA) cohort (n=349) (119) that were retrieved from the Human Protein Atlas (127). Information per sample includes sample ID, patient age, cancer stage, outcome, survival time, and quantified expression of select genes.

Sample ID	Age (Years)	Stage	Outcome	Survival (Days)	*HSPG2* (pTPM)	*KMT2C* (pTPM)	*MMP14* (pTPM)	*MMP16* (pTPM)	*MUC5AC* (pTPM)	*NCOR2* (pTPM)	*SPTBN2* (pTPM)	*TLL1* (pTPM)
TCGA-61-2088-01A	51	IIIC	alive	145	267.5	29.6	129.8	0	1	383.1	129.7	0.1
TCGA-36-1568-01A	52	IIIC	alive	875	38.1	18.8	71.4	0.1	0	119.9	108.5	0.1
TCGA-29-1776-01A	63	IIIC	alive	360	38.3	5.1	68.5	0.1	0	81.4	104.3	0.5
TCGA-24-1604-01A	66	IIIC	dead	2688	39.8	10.8	24.8	0	0.1	81.4	99.6	0
TCGA-24-1546-01A	46	IIIC	dead	1955	79.9	10.6	286	0.2	1	186	82.9	1.1
TCGA-25-1623-01A	71	IV	dead	565	85.2	8.5	185.5	0.1	0.4	109.4	76.8	1.1
TCGA-29-1710-01A	54	IIIC	dead	951	99	8.3	144	0.1	0	121.2	75.7	0.5
TCGA-59-A5PD-01A	55	IC	dead	624	73.6	13.7	49.6	0	0	148.8	71.6	0.2
TCGA-61-2098-01A	62	IIIC	alive	1993	98.7	14.6	67.4	0.4	22	230.8	71.1	0.4
TCGA-24-1423-01A	61	IIIC	alive	190	53	9.1	68.9	0.1	0.1	83	69.7	0.1
TCGA-59-2354-01A	63	IIIC	dead	1046	67.6	9.4	196.2	0.2	0	174.5	69.4	0.6
TCGA-24-1469-01A	71	IIIC	alive	277	56.3	7.5	58.2	0	0.1	127.9	65.2	0.2
TCGA-13-0714-01A	55	IV	dead	189	75.5	18.5	85	0.2	0	142.5	63.6	0.2
TCGA-24-1549-01A	58	IIIB	dead	1721	110.1	12.7	85.3	0	0	258	63.1	0
TCGA-24-2027-01A	51	IV	dead	3337	44.9	11.5	81.4	0.5	2	128.3	61.9	0
TCGA-25-1632-01A	68	IV	dead	1799	56.6	15.2	51.4	0.1	0.1	59.2	60.4	0
TCGA-36-1576-01A	76	IIIC	alive	915	109.4	6.2	295.9	0.1	0.1	128.7	58.7	0.4
TCGA-30-1862-01A	65	IV	dead	186	107	13.4	153.1	0.1	0.1	106.1	58.1	0.5
TCGA-04-1332-01A	70	IIIC	dead	1247	259.3	17.7	444.4	0.5	0	239.3	57.5	0.1
TCGA-29-1711-01A	45	IIIC	alive	1053	57.3	15.3	113.3	0.1	0	43.5	56.9	0.2
TCGA-04-1362-01A	59	IIC	dead	1348	33.5	14.8	18.7	0.1	0.2	110.6	56.5	0.1
TCGA-29-1694-01A	45	IIIC	dead	1187	49.1	9.8	112.4	0.1	0	65.1	56.4	0.2
TCGA-23-2078-01A	66	IIIC	alive	2661	123.6	15.4	186.2	0.5	0.1	167.9	56.1	0.2
TCGA-61-2109-01A	40	IIIC	dead	629	92.4	15.3	34.9	0	0	71.3	55.8	0.3
TCGA-13-0897-01A	54	IIIC	dead	2182	16.9	4.8	55.8	0.1	0	34.1	54.6	0
TCGA-24-2289-01A	68	IV	dead	2049	136.9	10	450.2	0.2	0	166.4	53.7	0.7
TCGA-30-1857-01A	64	IV	dead	8	23.3	9.5	16.7	0.2	0	31.5	51.2	0.4
TCGA-24-1427-01A	58	IIIC	alive	147	108.9	10.2	255.5	0.3	0	193.8	51	0.5
TCGA-61-1910-01A	56	IIC	alive	1127	83.3	22.4	33.2	0.1	0	127.4	50	0
TCGA-61-2097-01A	71	IIC	alive	1844	71.7	29.1	143.3	0.3	2.3	130.8	50	0.1
TCGA-24-2035-01A	65	IIIC	dead	857	103.8	8.4	91.6	0.5	0	85.2	49.8	0.9
TCGA-24-1434-01A	59	IIIC	dead	568	136.4	11.3	366.5	0.3	0	178.1	49.7	0.4
TCGA-57-1584-01A	47	IIIC	alive	643	23.4	16.8	40.2	0.1	0.1	46.9	49.4	0
TCGA-VG-A8LO-01A	55	IV	dead	24	91.6	6.9	57.6	0	0.5	127.7	48.3	0.1
TCGA-57-1993-01A	56	IIIC	alive	763	72.6	22.1	88.1	0.1	0.1	250.2	47.8	0
TCGA-24-1928-01A	77	IIIC	dead	336	79.8	15.3	183.7	0.3	0	119	47.7	0.4
TCGA-25-1626-01A	65	IIIC	dead	518	279.3	12	563.6	0.5	0.2	217	47.1	1.9
TCGA-04-1514-01A	45	IIIA	dead	1720	63.2	28.5	59.3	0.6	0.5	229.6	46.8	1.1
TCGA-24-1103-01A	50	IIIC	dead	1646	13.5	8.8	5.9	0	0.1	110.9	45.8	0
TCGA-24-1563-01A	66	IIIC	dead	1451	38	7.4	217.2	0.4	0	136.5	45.6	0.6
TCGA-25-1318-01A	54	IIIC	dead	1064	63.6	17.4	61	0.1	0	147.6	45.2	0.2
TCGA-61-1998-01A	48	IIIC	alive	168	141.8	8.3	129.1	0.2	0.1	100.1	44.9	0.2
TCGA-61-2113-01A	53	IIC	dead	676	127.1	20.9	194.6	0.2	1.7	174	44.5	0.5
TCGA-25-2042-01A	60	IIIC	dead	396	251.1	16.8	450.8	0.7	0.1	217.7	44.2	1.5
TCGA-61-2104-01A	53	IIC	alive	2338	94.6	22.9	59	0.2	0	100.7	43.8	1.1
TCGA-25-2404-01A	38	IIIC	dead	883	68.2	6.3	128	0.2	0	128.4	43.3	0.2
TCGA-24-2026-01A	79	IIIC	dead	1059	76.3	9.8	45.8	0	0	51.2	42.6	0.1
TCGA-24-2293-01A	47	-	dead	506	143.7	11.8	327.2	0.3	0	166.1	42.4	0.8
TCGA-23-1027-01A	48	IIIC	dead	976	73.2	4.1	78.6	0.1	0	55.4	41.9	0
TCGA-24-2254-01A	66	IIIC	dead	1736	95.1	21.2	150.3	0.6	0	104.7	41.7	0.3
TCGA-09-0364-01A	80	IIC	dead	887	77.4	11.6	267.6	0.2	0.1	448.7	41.4	0.2
TCGA-24-1467-01A	51	IIIC	dead	3224	57.4	12.8	28.4	0.1	0	122.9	40.7	0.2
TCGA-23-1110-01A	42	IIIC	alive	1658	86.8	8.9	50.5	1.2	0	201.6	40.6	0.1
TCGA-24-1846-01A	45	IIIC	alive	133	20.4	13.3	80.6	0	0	79.1	39.8	0.2
TCGA-24-1425-01A	45	IIIC	alive	181	235.4	22.9	451.6	0.5	0	231.5	39.5	0.9
TCGA-57-1585-01A	57	IIIC	dead	53	147.3	11.2	371.8	0.3	0	143.4	39.3	1.6
TCGA-61-1918-01A	45	IV	dead	479	59.2	10.2	47.6	0	0	86	39	0
TCGA-24-1567-01A	54	IIIB	dead	524	127.5	9.4	145.2	0.1	0.2	127.4	38.4	0.1
TCGA-24-1435-01A	57	IIIC	dead	1324	55.4	13.5	260.6	0.3	0	150.7	38.1	0.2

TCGA-23-2084-01A	45	IV	dead	1516	42.3	10	74.2	0.1	0	82.5	37.6	0
TCGA-29-1785-01A	55	IIIC	dead	1104	21.1	7.2	68.7	0	0	74.6	37	0
TCGA-24-1844-01A	64	IIIC	alive	113	55.4	10.2	40.2	0.2	0.1	98.3	36.6	0.2
TCGA-5X-AA5U-01A	61	IIC	alive	361	293.3	19.3	78.1	2.4	0	227.9	36	0.1
TCGA-29-2414-01A	75	IIIC	dead	2621	96.8	8.4	208.2	0.1	0	97.1	35.9	0.3
TCGA-29-1705-01A	47	IIIC	dead	555	58.8	19.2	162.1	0.3	0	141.3	35.9	0.3
TCGA-20-1682-01A	56	IIIC	alive	837	31.3	15.1	91.3	0.1	0	110.7	35.9	0.2
TCGA-13-0726-01A	55	IIIC	dead	949	31.9	0.6	19.7	0.1	0	32.8	35.7	0.1
TCGA-09-2045-01A	50	IV	dead	1069	63.7	21.7	39.7	0.1	0	89.1	35.6	0.1
TCGA-61-1900-01A	51	IIIB	alive	176	76.5	17.9	56.5	0.3	0.2	85.4	35.3	0.1
TCGA-25-1633-01A	64	IIIC	dead	1891	138.1	10.9	358.9	0.3	0.1	187.3	34.4	0.5
TCGA-13-1505-01A	63	IIIC	alive	1998	67.6	12	106.3	0.2	0	230.2	34.2	0.7
TCGA-61-2111-01A	61	IV	alive	3825	133.3	26.8	92.5	0.3	0.1	65.3	34.1	0.5
TCGA-24-1418-01A	68	IIIC	alive	243	131	14.4	162.2	0.1	0.1	179.4	34.1	0.6
TCGA-25-2409-01A	71	IV	dead	821	99	8.6	90.4	0.1	0	83.4	33.9	0.2
TCGA-29-A5NZ-01A	66	IIIC	dead	1088	265.6	27	443.5	0.6	0	259	33.8	0.3
TCGA-57-1582-01A	50	IIIC	dead	731	90.2	11.3	55	0.2	0	79.4	33.7	1.6
TCGA-23-1023-01A	65	IIIC	alive	1233	107.3	10.4	88.9	0.1	0.1	156.5	33.6	0.3
TCGA-29-1783-01A	58	IIIC	alive	220	151.2	11.3	121.6	0.2	0.2	184.8	33.5	0.2
TCGA-09-1662-01A	58	IV	dead	2717	45.2	11.7	45.9	0.1	0	69.6	33.4	0.1
TCGA-25-2398-01A	71	IIIC	dead	1369	131.8	11.6	432.1	0.5	0.1	206.1	31.9	0.6
TCGA-25-1627-01A	73	IIIC	dead	394	24.3	24.7	125.2	0.1	0.4	116.4	31.6	0.1
TCGA-29-2427-01A	60	IIIC	alive	1900	23.4	4.3	78.5	0.1	0	45.8	31.5	0.2
TCGA-29-1763-01A	43	IIC	alive	2032	115.2	22.1	38.5	0.3	0	105.1	31.4	0.2
TCGA-24-1544-01A	71	IIIC	dead	820	73.9	9.6	14	0.1	0.4	127.4	31.4	0
TCGA-24-2036-01A	50	IIIA	dead	1947	92.6	14.5	80.2	0.2	0.1	88.1	31.3	0.1
TCGA-25-1631-01A	73	IIIC	dead	9	33	13.3	46.7	0.1	1	146.2	31.3	0.1
TCGA-24-1424-01A	67	IIIC	alive	183	110	18.8	124.3	0.1	0.1	122.8	31.1	0.2
TCGA-61-2101-01A	55	IIIC	dead	1688	37.1	11.5	109.5	0.4	1.3	78.4	30.3	0.5
TCGA-04-1519-01A	48	IIIC	alive	24	17.9	11.2	39	0.1	0.2	69.1	30.3	0
TCGA-24-2290-01A	56	IIIC	dead	1102	29.5	18	58.1	0.1	0	134.2	30.2	0.2
TCGA-25-1329-01A	76	IIIC	dead	457	48.8	12.6	79.3	0.2	0	108	30.1	0.6
TCGA-13-0800-01A	52	IIIC	alive	2661	37.8	6.2	42.1	0.3	0	76.3	29.9	0.1
TCGA-31-1946-01A	30	IIIC	alive	918	84.5	9.2	188.1	0.2	0	101.8	29.7	0.2
TCGA-31-1953-01A	52	IIIC	alive	204	105.6	13.4	195	0.1	0	109.2	29.6	0.1
TCGA-29-1691-01A	51	IIIC	dead	1470	65.5	6.6	59.7	0.1	0	78	29.5	0.1
TCGA-29-1690-01A	66	IIIC	dead	1448	22.6	4.3	50.5	0.1	0.2	123.8	29.5	0
TCGA-24-2281-01A	68	IIA	alive	1357	39.9	7.1	74	0	0	131.1	29.3	0
TCGA-25-1628-01A	67	IIIC	dead	627	277.5	11.5	460.5	1.8	0.1	177.7	29.1	1.4
TCGA-25-1322-01A	62	IV	dead	91	72.3	16.8	55.2	0.1	0	135	29.1	0.3
TCGA-31-1944-01A	47	IIIC	alive	1386	84.4	10.2	66.8	0.1	0	131	29	0
TCGA-13-0797-01A	49	IIIC	alive	2121	34.5	20.5	8.8	0.1	0	59.4	29	0
TCGA-04-1361-01A	57	IIIB	alive	989	17	9.1	20.7	0.2	0.1	37.2	29	0.1
TCGA-31-1959-01A	49	IV	alive	67	135.3	11	311.8	0.3	0.1	109.3	28.9	0.6
TCGA-57-1994-01A	63	-	alive	761	159.2	19.6	71.3	0.1	0.1	142.3	28.6	0.4
TCGA-04-1530-01A	68	IIIC	dead	3622	211.5	9.6	192.2	0.3	0	192.8	28.5	0.3
TCGA-24-1431-01A	67	IIIC	dead	583	34.5	5.5	13.2	0	0	29.6	28.2	0.6
TCGA-24-1847-01A	45	IV	alive	343	56.2	10.8	46.3	0.1	0	91.6	28	0.1
TCGA-23-1120-01A	60	IIIC	alive	130	37.3	14.7	48.9	0.2	0	60	28	0.1
TCGA-25-1328-01A	38	IIIC	dead	2009	118	15	143.2	0.1	0	159.7	27.6	0.4
TCGA-25-1313-01A	62	IV	dead	820	18.4	31.1	37.3	0.1	0	72	26.6	0.1
TCGA-24-2261-01A	76	IIIC	dead	24	82.7	23.2	68.6	0.1	0.1	198.2	26.4	7.2
TCGA-30-1718-01A	44	IIIC	dead	1579	30.9	7.2	130.1	0.3	0	46.6	26.4	0.6
TCGA-24-1474-01A	57	IIIC	dead	676	88.5	22.7	213.9	0.2	0	89.2	26.2	0.3
TCGA-24-1105-01A	36	IIIC	dead	1442	71.8	2.2	42.8	1.9	0	124.1	26.2	0.2
TCGA-04-1648-01A	57	IIIC	dead	871	17.6	11.9	22.7	0.1	0	47.8	26.1	0
TCGA-13-1411-01A	81	IIIC	dead	531	90.2	10.6	281.1	0.2	0	111.3	25.9	0.4
TCGA-61-1724-01A	47	IIIC	dead	637	71.2	8	97.3	0.2	0	81.1	25.8	0.2
TCGA-61-2008-01A	40	IIC	alive	932	37.1	6.3	52.3	0.5	0	99.5	25.7	0.3
TCGA-20-1687-01A	46	IV	alive	81	42	7.9	25.8	0.1	0	62.4	25.6	0.1
TCGA-09-1673-01A	50	IV	alive	92	59.6	31.5	19.2	0.9	0	75.3	25.5	0.2
TCGA-24-1557-01A	49	IIIC	dead	1213	27.4	12.1	33.4	0.6	0.4	116.9	25.4	0.8
TCGA-04-1536-01A	60	IV	dead	885	5.9	12.6	14.7	0	0	32.2	25.4	0.1
TCGA-25-1320-01A	65	IIIC	dead	1155	101.5	13.2	247.8	0.8	0	179.3	25.1	2.9
TCGA-23-1111-01A	63	IIIC	alive	98	46.7	12.8	35.8	0.2	0.2	86.1	25.1	0.6
TCGA-23-1022-01A	67	IIIC	dead	1511	43.7	5	27.4	0.1	0	41.2	25.1	0.2
TCGA-25-1321-01A	65	IIIC	dead	1033	31.2	16.9	58.8	0.1	0	76.6	25	0.3
TCGA-25-1317-01A	66	IIIC	dead	61	42.9	12.7	63.9	0.1	0.1	113.6	24.7	0.2
TCGA-13-1408-01A	59	IIIC	dead	1680	101.3	7	151.4	0.3	0	63.8	24.6	0.5
TCGA-24-2297-01A	56	IIIC	dead	1699	60.6	8.4	27.7	0.3	0	48.9	24.6	0.8

TCGA-09-0369-01A	56	IIIC	dead	1082	140.9	3.9	140.3	0.2	0	140.6	24.2	0.1
TCGA-61-2003-01A	53	IIIC	alive	122	197	12.3	307.8	0.2	0.1	163.7	24.1	0.2
TCGA-25-1635-01A	71	IIIC	dead	1583	48.7	14.4	160.7	0.2	0	165	23.9	1
TCGA-20-0987-01A	61	IIIC	dead	701	30.5	3.6	19.2	0	0	39	23.6	0
TCGA-24-1430-01A	68	IIIC	dead	863	68.7	15	57.5	1.1	0	100	23.3	0.2
TCGA-36-1574-01A	48	IIIC	alive	686	46.9	7.3	131.2	0.2	0	42.6	23.1	0.1
TCGA-13-1492-01A	66	IIIC	dead	3819	35.2	15.4	91.6	0	0	94.6	23.1	0.1
TCGA-24-1104-01A	56	IV	dead	1933	36.4	17.1	15.9	0.1	0	58.1	23	0.1
TCGA-31-1951-01A	58	IIIC	alive	684	164.7	16.2	157.7	1.4	0	154.1	22.9	0.5
TCGA-25-2392-01A	75	IV	dead	31	13.9	14.8	39.6	0.3	0	137.9	22.9	0.1
TCGA-24-1553-01A	53	IIIB	dead	1767	50.4	6.8	52	0.1	0	90.5	22.8	0.1
TCGA-24-2271-01A	55	IIIC	dead	962	49.5	8.8	67.9	0.2	0	158.8	22.7	0.2
TCGA-13-1410-01A	57	IV	alive	2464	70.7	8.3	104.8	0.5	0	55.2	22.3	0.4
TCGA-61-1741-01A	76	IIIB	dead	1024	154.3	14.7	56.7	1.9	0.9	131.8	22.1	1.5
TCGA-20-1683-01A	65	IIIC	alive	772	22.7	7.4	26.8	1.8	0	150.3	22.1	0.1
TCGA-25-1316-01A	55	IIIC	dead	1279	41.5	7.9	64.3	1.9	0	79.4	22	0
TCGA-25-2399-01A	80	IIIC	dead	608	140.3	13.1	166.2	0.1	0	141.6	21.8	0.3
TCGA-04-1651-01A	53	IIIC	dead	1102	8.2	14.8	7.9	0	0.2	28.9	21.7	0.3
TCGA-OY-A56Q-01A	78	IIA	alive	576	111.7	30.8	29.4	0	0	126.8	21.5	0
TCGA-WR-A838-01A	72	IIIC	dead	304	54.7	15.5	55.8	0.2	0	82.5	21.5	0.4
TCGA-13-1501-01A	50	IV	dead	1314	13.4	8.5	20.4	0.1	0	31.1	21.4	0.2
TCGA-13-0891-01A	73	IV	dead	3128	13.9	7.6	19.1	0.2	0	30.6	21.3	0.3
TCGA-36-1569-01A	52	IIIC	alive	885	112.2	14	213.9	0.3	0	280.4	21	1
TCGA-23-1123-01A	59	IIIC	dead	1018	73.5	12	85.4	0	0.2	87.5	20.9	0.2
TCGA-29-1774-01A	82	IIIC	alive	527	20.1	8.6	86.8	0.5	0.3	81.7	20.8	0
TCGA-61-2002-01A	46	IIIC	alive	547	50.1	7.8	125.5	0.1	0	119.3	20.7	0.2
TCGA-29-2428-01A	58	IIIC	alive	1372	26.3	8.7	51.6	0.1	0.3	140.6	20.7	0.1
TCGA-59-2351-01A	51	IIIC	alive	3532	45.1	8.6	55.8	0.2	0	90.9	20.2	0.1
TCGA-23-1024-01A	52	IV	alive	468	38.2	5.9	11.8	0	0	61.7	20.2	0.1
TCGA-04-1364-01A	61	IIIC	dead	1024	18.7	4.2	23.8	0.1	0.2	77.2	20.2	0
TCGA-24-1422-01A	82	IIIC	dead	23	59.1	13.2	194.8	0.5	0.1	87.5	19.8	0.7
TCGA-09-0366-01A	55	IIIC	dead	1757	68.2	11	22.3	0.1	0.1	110.8	19.5	0
TCGA-61-1995-01A	43	IIIC	alive	61	145	16	74	0	0	83.8	19.4	0
TCGA-57-1586-01A	66	IIIC	alive	679	47.5	11	35.3	0.1	0.2	162.2	19.4	0.8
TCGA-24-1562-01A	67	IIIC	dead	1384	116.4	10.2	101.8	0.1	0	155.2	19.1	0.3
TCGA-24-1550-01A	49	IIIC	dead	1249	98	4.7	320.5	0.2	0.1	176.3	18.9	1.2
TCGA-04-1343-01A	72	IV	dead	361	65.7	3.8	40.2	0.1	0.2	69.7	18.9	0.1
TCGA-29-1695-01A	62	IIIC	dead	1229	127.8	17.1	267.7	0.5	0.1	84	18.8	0.7
TCGA-24-1551-01A	53	IIIC	dead	1579	46.1	4.8	42.4	0.1	0	72.1	18.8	0.3
TCGA-13-0885-01A	70	IIIC	alive	3388	25.1	8.1	18.4	0	0	51.5	18.7	0
TCGA-13-1497-01A	47	IIIC	alive	3871	25.1	7.2	68.2	0.1	0	60.3	18.6	0
TCGA-24-2267-01A	58	IIB	dead	1446	50.3	17.1	31.6	0.1	0	59.7	18.5	0.7
TCGA-23-1122-01A	53	IIIC	dead	1189	81.5	9.1	91.9	0.5	0	79.8	18.1	1.2
TCGA-13-1511-01A	52	IV	dead	1650	106.7	17.5	88.7	0.2	0	101.5	18	0.2
TCGA-61-2092-01A	57	IIIC	alive	1573	22.1	7.6	4.2	0.1	1.3	31.3	18	0
TCGA-59-2355-01A	58	IV	dead	65	37.7	8.7	89.2	0.1	0	75.2	17.7	0
TCGA-24-1428-01A	50	IIIC	alive	529	26.3	4.5	39.8	0.1	0	67.8	17.6	0.1
TCGA-25-1319-01A	73	IIIC	dead	1977	51.3	12.9	51.6	0.1	0	99.9	17.4	0
TCGA-24-1419-01A	62	IIIC	alive	239	30.7	15.4	64.6	0.1	0	119.6	17.2	0
TCGA-24-1413-01A	51	IIIC	alive	192	25.9	17.8	34.9	0	0	80.1	17.2	0.1
TCGA-09-0367-01A	67	IIIC	dead	547	13.1	5.3	6.7	0	0	20.3	17.1	0.1
TCGA-36-1571-01A	53	IIIB	dead	695	65.9	12	112	0.1	0	136.3	17	0
TCGA-25-1630-01A	73	IIIC	dead	1162	31.4	7	60.3	0.1	0.1	37.8	17	0.1
TCGA-13-1506-01A	45	IIIC	dead	1039	11.9	5.8	15.9	0	0.1	68.4	17	0.1
TCGA-24-1924-01A	65	IIIC	dead	919	17	9.1	27.7	0	0	43.3	16.5	0.1
TCGA-57-1583-01A	57	IIIC	dead	346	51	7.8	75.2	0.2	0	93.2	16.3	0.2
TCGA-59-2348-01A	59	IIIC	alive	5481	51.7	15.1	72.6	0.1	0	189.9	16.2	0.2
TCGA-24-1417-01A	54	IV	alive	238	78.6	15	134.4	0.1	0	88	16	0.5
TCGA-25-2396-01A	71	IIIC	dead	92	36	6.8	71.5	0	0	44.6	16	0.2
TCGA-59-2363-01A	40	IIIA	alive	165	27.4	14.3	41.9	0.1	1.4	151.3	16	0.2
TCGA-13-1499-01A	56	IIIC	alive	3500	26.5	7.3	59.8	0.2	0.2	44.3	16	0.3
TCGA-13-1407-01A	51	IIIC	alive	2534	21.4	4.9	20.5	0.1	0	43.9	15.9	0.1
TCGA-24-2298-01A	55	IIIC	dead	1620	45	12.6	128.5	0.2	0	94.6	15.8	0.4
TCGA-23-1030-01A	64	IIIC	alive	886	85.9	7	38.5	0.3	0	70.7	15.7	0.1
TCGA-24-1558-01A	73	IIIC	dead	594	120	16.4	166.9	0	0	139.1	15.6	0
TCGA-24-2024-01A	72	IIIC	dead	1769	44.2	9.9	88.7	0.3	0	66	15.5	0.1
TCGA-13-0720-01A	48	IIIC	dead	1355	17	9.4	6.2	0	0	38.4	15.3	0
TCGA-10-0928-01A	71	IIIC	dead	563	34.3	10.2	24.9	0.1	0.1	34.8	15.2	0
TCGA-13-1483-01A	61	IIIC	dead	895	25.6	9.2	51.6	0.1	0	56.9	15.1	0.1
TCGA-23-1109-01A	62	IIIC	dead	1562	94	9.1	74.3	0.6	0	73.4	14.9	0.2

TCGA-13-1403-01A	48	IIIC	dead	2345	49.8	8.3	52.8	0.2	0	56.2	14.8	0.1
TCGA-29-1703-01A	56	IIIC	dead	1815	35.9	5.4	62.1	0.3	0	54.3	14.8	0.1
TCGA-13-1405-01A	49	IV	dead	868	151.1	7.6	110.3	0.3	0	90.1	14.6	0.2
TCGA-24-1850-01A	72	IIIC	alive	168	56.4	7.2	188.7	0.1	0	83.9	14.4	0.2
TCGA-09-1669-01A	54	IIIA	alive	928	22.2	8.8	39.6	0.1	0.2	59.5	14.4	0
TCGA-13-0924-01A	45	IV	alive	2614	14.8	7.9	32.7	0	0	61	14.2	0.1
TCGA-13-1487-01A	74	IV	dead	681	38	6.3	71.7	0.2	0	62.2	14.1	0.3
TCGA-24-1565-01A	74	IIIC	dead	312	4.9	6.9	10.6	0	0	91.4	14.1	0
TCGA-61-1728-01A	59	IV	alive	848	30	6.5	22.2	0	0	54.1	13.9	0.2
TCGA-25-1326-01A	61	IIIC	dead	1249	57.3	10.9	152.4	0.2	0	97.7	13.8	0.3
TCGA-24-2038-01A	68	IIIA	dead	1354	36.3	8.5	18.8	0.2	0.1	98.7	13.8	0.1
TCGA-23-1809-01A	63	IIC	alive	16	90.4	14	276	0.3	0.2	454.1	13.6	0
TCGA-13-A5FT-01A	67	IIIC	alive	2143	36.2	13.8	27.7	0.1	0.1	205.5	13.6	0.1
TCGA-13-1477-01A	49	IV	dead	1662	13.3	5.6	37.5	0.1	0	32.9	13.6	0.1
TCGA-24-2288-01A	70	IIIC	dead	25	47.9	16.3	102.9	0.1	0	85.6	13.5	0.5
TCGA-24-1552-01A	77	IIIC	dead	1259	44.8	7.2	21.9	0.2	0	41.5	13.5	1.1
TCGA-13-1485-01A	48	IV	dead	629	54.8	8.1	268.8	0.2	0	101.5	13.4	0.2
TCGA-04-1331-01A	78	IIIC	dead	1336	37.4	12.1	186.7	0.2	0	104.4	13.4	0.2
TCGA-59-2352-01A	78	IIIC	dead	286	152.1	11.1	157.7	0.1	0	105.7	13.3	0.1
TCGA-25-1323-01A	72	IIIC	dead	395	107.8	5.9	68.2	0.2	0.4	112.7	13.3	0.1
TCGA-24-1842-01A	49	IIIC	alive	253	54	7.2	47.3	0	0.3	170.1	13.3	1.4
TCGA-09-1666-01A	57	IIIC	alive	1752	10.8	15.4	26.2	0.2	0	26.6	13.2	0.1
TCGA-13-0730-01A	71	IIIC	dead	542	50.4	8.2	260.8	1.4	0.2	106.3	13	2.1
TCGA-24-1416-01A	34	IV	alive	194	19.6	7.7	17.5	0.1	0.1	83.3	13	0.1
TCGA-29-1768-01A	50	IV	dead	952	17.2	3.9	120.3	0.2	0	26.4	13	0.6
TCGA-36-1581-01A	63	IIC	alive	751	40	9.7	141.2	0.1	0	134.1	12.8	0.1
TCGA-31-1950-01A	76	IIIC	alive	571	42.6	11.1	19.8	0	0.1	95	12.7	0
TCGA-29-1693-01A	72	IIIC	alive	3096	35.4	3.5	60.8	0.3	0	58	12.7	0.2
TCGA-13-0923-01A	74	IIIC	alive	2547	15.9	5.7	39.5	0	0	19.5	12.7	0.1
TCGA-36-1570-01A	49	IIIC	alive	655	30.8	8.6	19.4	0.1	0	56.4	12.6	0.1
TCGA-24-1843-01A	66	IIIC	alive	106	41.4	6	37.2	0	0	72	12.5	0
TCGA-04-1338-01A	78	IIIC	alive	1418	11.9	16	64.9	0.1	0	32.7	12.4	0.6
TCGA-24-2023-01A	54	IIIA	dead	1364	48.1	9	16.1	0	0	51.4	12.3	0
TCGA-13-1488-01A	59	IV	dead	2154	7.1	4	12.2	0.1	0	27.8	12	0.1
TCGA-24-0966-01A	78	IIIC	alive	232	3.7	3.7	12.9	0	0	12.6	12	0
TCGA-23-1116-01A	83	IIIC	dead	592	49.7	4.9	105.1	0.2	0	66.6	11.9	0.1
TCGA-13-0906-01A	50	IIIC	alive	3619	34.6	8.8	41.7	0.1	0	58	11.7	0.7
TCGA-20-1686-01A	75	IIIC	alive	89	8	12.5	9.7	0.1	0.2	14.7	11.7	0.1
TCGA-09-2054-01A	58	IIIC	dead	637	124	19.9	159.6	0.2	0.1	103.9	11.6	0.4
TCGA-13-0883-01A	61	IIIC	dead	2097	79.8	12.4	330.7	0.5	0	124.1	11.6	0.2
TCGA-25-1870-01A	59	IIIC	dead	455	41.1	19.5	50.4	0.1	0.1	112.6	11.3	0.4
TCGA-29-1762-01A	59	IV	dead	2634	37.6	11.2	44.8	0.1	0	56	11.1	0.2
TCGA-04-1542-01A	52	IIIB	dead	2561	15.9	3.1	13.7	0	0	39.7	11	0
TCGA-29-1696-01A	43	IIIC	dead	1032	4.2	4.3	3.5	0	0	19.5	11	0.1
TCGA-24-2033-01A	87	IIIC	dead	562	56.3	7.5	58.4	0.4	0.2	111.9	10.8	0.1
TCGA-10-0931-01A	44	IIIC	dead	1000	8.1	9.3	2.7	0	0	26.9	10.8	0.1
TCGA-25-1312-01A	69	IV	dead	31	100.4	11.2	32.3	0.3	0	103.9	10.7	0.2
TCGA-23-2077-01A	45	IIIC	alive	3525	75.8	9	54	0.2	0	53.3	10.5	0.1
TCGA-36-1577-01A	43	IIC	alive	783	50	7.1	87.4	0.3	0.3	107.3	10.5	0.1
TCGA-25-1315-01A	50	IIIC	dead	1583	46.6	11	54.3	0.1	0	85.4	10.5	0
TCGA-61-2102-01A	74	IIIC	dead	197	88.1	11.2	466.5	0.3	0.2	259.9	10.4	0.3
TCGA-29-2425-01A	60	IIIC	alive	1977	70.1	5.2	24.8	0.1	0	54.6	10.4	0.1
TCGA-61-2009-01A	65	IIIC	alive	1212	22.7	3.7	112.4	0.3	0.4	62.3	10.3	0.2
TCGA-61-1737-01A	42	IV	alive	1364	53.5	15.1	208.3	0.4	0	79.8	10.2	0.2
TCGA-13-2060-01A	51	IV	alive	2369	25.8	9.1	35.2	0.1	0	27.4	10.2	0.1
TCGA-13-0888-01A	78	IIIC	dead	2811	64.9	11.4	47.7	0.3	0.1	105.5	10.1	0.1
TCGA-09-2051-01A	42	IIIC	alive	1919	30.8	11.3	32.2	0	0	59.2	10.1	0
TCGA-29-1701-01A	56	IIIC	dead	515	13.1	2.2	34	0	0	15.3	9.9	0.2
TCGA-30-1861-01A	74	IIIC	dead	1058	109.4	19.8	238.5	1.5	0	88.7	9.8	6.2
TCGA-24-1923-01A	51	IIIC	dead	690	32.5	7.5	28.6	0.2	0	61.9	9.8	0.1
TCGA-61-1725-01A	40	IIIC	alive	956	9.3	3.3	39.3	0.1	0	20.5	9.8	0.1
TCGA-30-1866-01A	61	IV	dead	1114	6.5	5	40.1	0	0	12.3	9.8	0.2
TCGA-13-1489-01A	70	IIIC	dead	2553	4.9	8.8	13.5	0	0.2	21.2	9.8	0.1
TCGA-59-2350-01A	44	IV	dead	679	37.5	9	78.5	0.1	0	46.7	9.7	0.1
TCGA-13-1510-01A	62	IIIC	dead	1359	6.3	8.5	24.6	0.1	0	44.2	9.5	0.1
TCGA-13-1512-01A	49	IIIC	alive	442	19.4	6.7	56.1	0.1	0	40	9.4	0.2
TCGA-09-1670-01A	57	IIIA	alive	547	28.9	11.1	29	0.1	0	88.4	9.2	0
TCGA-13-0920-01A	65	IIIC	dead	1484	14.1	3.8	26.1	0.2	0	24.9	9.2	0
TCGA-13-0762-01A	65	IIIC	alive	3400	28.8	8.3	22.6	0.1	0	27.2	9.1	0
TCGA-10-0938-01A	80	IIIC	dead	636	9.6	5.4	6.1	0	0	41.2	9.1	0.3

TCGA-04-1356-01A	62	IIC	dead	1499	17.1	7.2	87.5	0.2	0	30.8	9	0.1
TCGA-23-1119-01A	64	IIIC	alive	3953	8	8	18.9	0.1	0	18.2	9	0.1
TCGA-13-0886-01A	67	IIIC	alive	4665	29.3	9.7	76.5	0.2	0	48.5	8.9	0.4
TCGA-04-1341-01A	85	-	alive	33	16.4	5	42.5	0	0	66.3	8.9	0
TCGA-25-1877-01A	81	IIIC	dead	730	5.2	2	100.7	0.3	0	10.6	8.9	1.2
TCGA-13-0725-01A	44	IIIC	dead	377	16.5	3.9	27.3	0.3	0	25.6	8.8	0.1
TCGA-13-0916-01A	49	IIIC	alive	1785	46.5	8.1	29.3	0.1	0	84.3	8.6	0
TCGA-10-0937-01A	44	IIIC	dead	608	4.9	4.2	9.6	0	0	14.9	8.6	0.1
TCGA-24-1845-01A	42	IIIC	alive	116	8.9	5.5	7.5	0	0	32.6	8.5	0
TCGA-36-1580-01A	82	IIIC	dead	737	42	9.9	164.9	0.3	0	88.9	8.4	0.7
TCGA-23-1118-01A	45	IIIC	alive	2616	19.2	8.5	27.4	0.1	0	22.3	8.4	0.1
TCGA-29-1697-01A	62	IIIC	dead	949	58.5	7.9	23.1	0.2	0	79	8.3	0
TCGA-30-1860-01A	58	IIIC	dead	1366	8.6	6.1	56.5	0.1	0	32.7	8.3	0.1
TCGA-13-0887-01A	42	IIIC	dead	2028	11	4.3	16.5	0	0	17.9	8.1	0
TCGA-24-1560-01A	51	IIIC	dead	1341	43.7	9.9	40.4	0.1	0	50.7	8	0
TCGA-61-1919-01A	58	IIIC	dead	1161	25.5	5.1	135	0.2	0	54.6	8	0.3
TCGA-25-2401-01A	64	IIIC	dead	90	76.4	10.6	190.2	0.4	0	62.9	7.9	0.5
TCGA-30-1891-01A	61	IIIC	dead	914	23.6	6.8	88.1	0.1	0	52.2	7.9	0.8
TCGA-24-2280-01A	74	IIIC	alive	2143	174.7	16.2	234.2	1	0	103.1	7.8	0.3
TCGA-29-1688-01A	39	IIIC	dead	2400	15.3	7.3	38.1	0.1	0	46.8	7.8	0.2
TCGA-61-2110-01A	56	IIIC	dead	1354	48.2	14.5	27.1	0	0	53.7	7.7	0.1
TCGA-24-0968-01A	59	IIIC	dead	598	15.2	8.4	61	0.1	0	57.6	7.6	0.1
TCGA-29-1769-01A	40	IIIC	alive	699	11.9	6.4	26.7	0.1	0	20.3	7.6	0.2
TCGA-29-1784-01A	55	IIIC	alive	163	7	5.2	13.7	0	0	23.9	7.6	0
TCGA-04-1365-01A	87	IIIB	alive	947	18	8.3	57.2	0.1	0	60.5	7.5	0.1
TCGA-24-0979-01A	53	IV	dead	1264	53	8.2	10.5	0.2	0	58.4	7.4	0.1
TCGA-61-2000-01A	67	IIIC	alive	441	27.8	5.8	36.3	0	0	62.9	7.4	0.2
TCGA-24-1930-01A	53	IIIC	dead	2467	39.1	10.1	105.8	0.1	0	98.7	7.3	0.2
TCGA-25-2400-01A	76	IIIC	dead	1278	38.3	15.4	55.8	0.1	0.3	94.1	7	0
TCGA-25-2393-01A	81	IIIC	dead	1157	26	4.7	29.6	0	0	80.4	7	0.1
TCGA-09-2048-01A	63	IIIC	dead	138	18.5	2.2	11.9	0.1	0	28.1	7	0
TCGA-13-1507-01A	77	IIIC	dead	1993	6.9	1.3	56.8	0	0	13.6	6.9	0.3
TCGA-13-1404-01A	48	IIIC	alive	2469	22.2	6.9	65.8	0.1	0	29.1	6.8	0.3
TCGA-13-0804-01A	73	IIIC	dead	1073	16.4	6.1	22.9	0	0	38.4	6.8	0
TCGA-13-0727-01A	71	IIIC	dead	462	101.7	5.1	56.1	0.1	0.2	118.6	6.7	0.6
TCGA-20-0991-01A	78	IIB	alive	797	9	3.5	41.1	0	0	39	6.7	0
TCGA-29-1777-01A	47	IIIC	alive	374	25.6	3.7	65.2	0	0	21.8	6.4	0.3
TCGA-61-2012-01A	81	IIC	alive	932	49	7.6	33.6	0.1	0.2	61.5	6.3	0.1
TCGA-30-1714-01A	68	IV	dead	1158	2.8	2.8	6.6	0	0	9.3	6.3	0.1
TCGA-29-1778-01A	77	IIIC	alive	454	90.8	11.5	35.3	0.1	0.1	48.2	6.2	0.1
TCGA-24-1464-01A	70	IIIC	dead	379	5.6	7.3	14.2	0.1	0	39.4	6.2	0.2
TCGA-13-1509-01A	64	IV	alive	2438	9.8	6	63.3	0.1	0	21.8	6.1	0.2
TCGA-23-1028-01A	43	IIIC	alive	1503	5.1	4.5	7	0	0	15.3	6.1	0
TCGA-25-1634-01A	75	IIIC	dead	1091	48.6	5.9	37.4	0.1	0.1	80.9	6	0
TCGA-24-1603-01A	53	IIIB	dead	2742	27.5	10.5	27.1	0.1	0	47.8	6	0.1
TCGA-10-0933-01A	77	IIIC	dead	446	13.6	8.4	52.7	0.2	0	36.4	6	0
TCGA-29-1766-01A	74	IIIC	dead	1199	24.3	2.9	81.2	0.1	0	21.8	5.9	0.5
TCGA-30-1892-01A	52	IIIC	dead	1484	23	2	64.9	0.6	0	23.3	5.8	0.3
TCGA-13-0911-01A	55	IV	dead	1355	31.9	2.5	71.8	0.1	0	40.1	5.6	0.3
TCGA-61-1914-01A	65	IIIC	alive	1722	20.3	4.8	18.5	0	0	19.7	5.5	0.1
TCGA-24-2020-01A	67	IIIC	dead	4624	75.8	11.1	27.4	0.1	0.4	122.7	5.4	0
TCGA-04-1347-01A	81	IV	alive	1919	25.2	3.8	38.2	0.1	0	49.6	5.4	0
TCGA-61-1738-01A	60	IIIC	dead	1089	14.6	4.5	52.6	0	0	32.3	5.4	0
TCGA-13-0766-01A	42	IIIC	dead	1725	7.8	6.9	12.6	0	0	22.1	5.4	0.1
TCGA-23-1107-01A	59	IV	dead	9	5.4	3.1	3.9	0.1	0.1	16.9	5.3	0.1
TCGA-31-1956-01A	60	IIIB	alive	1342	36.5	5.7	156.3	0.1	0	46.5	5.2	0.2
TCGA-04-1655-01A	49	IIIB	dead	1380	22.4	4.8	10.9	0	0	48.7	5.2	0
TCGA-13-1496-01A	65	IIIC	dead	129	12.1	9	19.5	0	0	30.1	5.2	0.1
TCGA-30-1853-01A	58	IIIC	dead	1103	17.8	5.8	13	0.3	0	26.8	5.1	0
TCGA-24-1616-01A	56	IIIC	dead	1163	46.9	13.8	34.3	0	0	78	4.8	0
TCGA-23-1113-01A	48	IV	dead	949	32.4	7.9	52.6	0.1	0	68.2	4.8	0.3
TCGA-61-1911-01A	55	IIA	alive	1293	18.8	4.2	14.7	0	0	44.3	4.6	0.1
TCGA-24-0982-01A	77	IIIC	dead	679	34.5	7.2	42.1	0.3	0.4	36.9	4.4	0.4
TCGA-24-1471-01A	60	IIIC	alive	36	23.4	5.7	7.6	0	0	22.6	4.4	1.4
TCGA-24-1426-01A	43	IIIC	alive	163	53.1	4	32.9	0.8	0.2	54.5	4.3	0.1
TCGA-13-0724-01A	72	IV	dead	83	24.2	7.5	20.3	0	0.1	18.9	4.3	0.2
TCGA-13-0765-01A	50	IIIC	dead	1389	12.6	2.6	8	0	0	42	4.2	0.1
TCGA-61-1907-01A	63	IIIC	alive	952	7	1.6	12.1	0	0	29.8	4.2	0.1
TCGA-13-1409-01A	73	IIIC	dead	1742	25.3	6	46	0.3	0	51.3	4	0.1
TCGA-13-1495-01A	60	IIIC	dead	2749	9.1	7.7	32.5	0.1	0.1	33.8	4	0.1

TCGA-61-1733-01A	71	IIIC	alive	967	167.8	10.6	86.3	0.1	0	90.4	3.5	0.2
TCGA-04-1350-01A	46	IIIC	dead	1946	16.1	4.5	12.8	0	0	20.5	3.2	0
TCGA-29-1770-01A	54	IIIC	alive	741	46.6	7	91.2	0.6	0	53.2	3	0.3
TCGA-13-0768-01A	73	IIIC	dead	1784	40.7	5.8	159.8	0.2	0	47.4	2.8	0.8
TCGA-10-0927-01A	65	IIIC	dead	2490	34.5	6.3	19.5	0.1	0	62.2	2.4	0
TCGA-13-1498-01A	73	IIIC	dead	2012	15.5	4.8	43.4	0.1	0	33.3	2.3	0
TCGA-29-1761-01A	80	IIIC	dead	528	5.2	6.9	17.6	0	0	24.6	2.2	0
TCGA-13-0795-01A	66	IIIC	dead	619	10	8.4	18.7	0	0	16.8	2	1.1
TCGA-29-1781-01A	69	IIIC	alive	255	11.9	5.4	31.3	0.3	0	11.7	1	0.9
TCGA-61-1721-01A	38	IV	alive	338	35.5	5.3	77.8	0.4	0	74.3	0.9	0.4

Note: Data entries listed on the Human Protein Atlas version 24.0 as of 2 May 2025.

Table S5: Data corresponding to human ovarian cancer samples from the Human Protein Atlas validation cohort (n=81) (128) that were retrieved from the Human Protein Atlas (127). Information per sample includes sample ID, patient age, cancer stage, outcome, survival time, and quantified expression of select genes.

Sample ID	Age (Years)	Stage	Outcome	Survival (Days)	*HSPG2* (pTPM)	*KMT2C* (pTPM)	*MMP14* (pTPM)	*MMP16* (pTPM)	*MUC5AC* (pTPM)	*NCOR2* (pTPM)	*SPTBN2* (pTPM)	*TLL1* (pTPM)
DO46571	61	III	dead	243	62.7	8.5	168.5	0.4	2.9	39.4	89.7	1.2
DO46328	52	III	dead	972	17.8	18.9	72	0.1	0	38.6	38.4	0
DO46362	61	III	dead	1639	83.3	22.7	104.4	0.1	3.7	95.4	35.3	0.1
DO46344	46	III	dead	642	28.5	18.1	149.2	0.2	0	35.8	34.9	0.4
DO46533	46	III	alive	3667	64.6	25.1	80.2	0.7	7.5	40.4	34.1	0.3
DO46404	55	III	dead	690	11.3	19.1	21.8	0.1	1.1	24.5	30.2	0.1
DO46342	67	III	dead	1398	16.7	1.7	16.4	0.1	1.1	8.7	29.1	0
DO46354	73	III	dead	843	29	16.4	161.8	0.1	0.5	33.2	27.7	0.7
DO46336	70	IV	dead	664	55	7.5	62.3	0.7	0	34.4	27.6	0.3
DO46513	54	IV	dead	1883	15.1	23.3	33.4	0	0.1	24.9	26.6	0
DO46508	50	III	dead	2101	92.8	16.8	404	0.6	0	30.2	25.5	1.5
DO46576	75	III	alive	133	40.1	3.2	23.7	0.1	0	8	24.9	0
DO46560	45	IV	dead	286	121.6	8.8	189	0	0	40.3	24.6	0.2
DO46326	51	III	dead	701	33.1	10.6	141.2	0.2	0.4	43	24.6	2.4
DO46518	62	III	alive	2960	22.8	14.1	17.3	0	0	13.8	24.5	0
DO46346	54	III	dead	1187	26.6	13.1	146.1	0.2	0	36.3	24	0.2
DO46538	57	III	dead	1527	25.1	21.7	20.4	0	0	24.5	23.7	0.7
DO46327	54	III	dead	184	83.4	11	366.4	0.2	0	36.8	23.1	0.6
DO46382	62	IV	dead	311	66.2	16.1	400.9	1	0	73.3	22.1	2.8
DO46390	65	III	dead	374	18.1	10.9	2.7	0.1	0.1	5.1	22	0.2
DO46360	58	IV	dead	1759	33.1	8.6	64.6	0.1	0	19.6	21.2	0.1
DO46325	54	III	dead	477	29.8	11.5	117.1	0.2	0	21.1	20.3	0.5
DO46348	58	IV	dead	287	72.9	10.1	417.2	0.3	0.1	42.1	19.7	1.7
DO46468	67	III	alive	2776	72.6	12.4	72.1	0.1	0	70	18.5	0.1
DO46606	73	III	alive	804	34.7	10	61.5	0.1	0.2	20.4	17.9	0.2
DO46546	73	III	dead	1889	16.2	14.3	3.5	0	0.1	11.6	17.9	0
DO46591	58	III	dead	195	11.3	3.1	40.5	0.2	2.2	8.5	17.6	0.1
DO46568	66	III	dead	320	32.6	8.9	106.7	0.3	0.2	37.5	17.1	0.3
DO46329	52	III	dead	1699	21.4	7.4	72.6	0.1	3.7	23	17.1	0.1
DO46388	74	III	dead	413	18.4	11.4	167.6	0.1	0.2	17.3	17.1	0.3
DO46370	54	III	dead	1727	43.4	8.3	47.5	0.1	0.9	20.8	16.5	0.2
DO46412	64	III	dead	597	16.8	4.7	30.5	0	0.1	14.7	16	0
DO46408	56	III	dead	489	28.4	18.4	36.9	0.1	0	23.2	15.8	0.1
DO46428	59	IV	alive	2644	11.1	10.7	6.5	0	5.1	8.2	15.6	0
DO46566	78	III	dead	673	146.9	8.9	91.2	0.5	0.1	35.5	15.2	0.1
DO46364	45	III	dead	806	44.3	5.6	223.1	0.2	0	27.9	15.2	0.6
DO46372	39	III	dead	1326	31.2	4.6	21.3	0	0	16	15	0.1
DO46597	60	III	dead	393	127.8	19.8	160.7	0.3	0.1	79.4	14.5	0.5
DO46438	55	IV	alive	2227	51.9	11.8	42.4	0.1	0	19.9	14.3	0.1
DO46453	66	III	dead	3294	23.8	2.6	137.9	0.1	0	11.5	14.1	0.1
DO46330	60	III	dead	684	40	16.6	62.6	0.1	1.4	39.1	13.8	0.1
DO46551	58	III	dead	1055	51.6	9.1	43.5	0.1	0	19.1	13.5	0.2
DO46340	62	III	dead	616	54.7	15.4	294.2	0.7	0	36.1	13.3	1
DO46528	56	III	alive	6139	48.8	6.7	168.3	0	0	39.8	13.2	2.4
DO46523	52	IV	alive	1873	34.6	18.5	57.7	0	0	11.9	13.1	0.3
DO46333	58	III	dead	359	11.7	2.2	9.9	0	0	3.7	12.4	0
DO46376	58	III	dead	1430	87.7	6.9	81.8	0	0	16.7	12.3	0.1
DO46443	59	III	dead	2464	36	15.2	121.6	0.4	0	28.1	11.8	1
DO46493	62	IV	alive	839	14.2	5.9	29.7	0.1	0.3	19.3	11.3	0.1
DO46332	56	III	dead	689	44.5	11.5	344.4	0.2	0	56	10.7	1.1
DO46356	68	III	dead	911	16.3	7.5	10.9	0.1	1.5	9.7	10	0
DO46611	52	III	dead	734	9	34.4	4.6	0.1	0.6	10.2	9.1	0.1
DO46561	52	III	dead	195	90.5	29.9	111.2	2.1	0.1	53.7	8.8	0.2
DO46368	57	III	dead	2879	31.4	8.3	73.4	0.1	0	18	8.6	0.2
DO46380	51	III	dead	1423	11.3	8.4	15.1	0.1	0.1	9.8	8.6	0
DO46400	70	III	dead	719	17.1	16.6	35.8	0	0	14.4	8.5	0.3
DO46581	66	III	dead	408	13.7	5.2	26.3	0	0.1	8.8	8.5	0
DO46366	65	III	dead	2054	7	1	8.9	0	0	3.9	8.1	0
DO46586	55	III	dead	1019	26.3	5.8	21.3	0	0	5.6	8	0

DO46334	77	III	dead	496	44.1	8	147.7	0.1	0	19.8	7.9	1.3
DO46386	49	III	dead	1162	17.8	9	72.3	0.1	0	12.7	7.9	0.8
DO46436	58	III	alive	3193	43.4	9	51.9	0.3	0.1	11.1	7.5	0.1
DO46378	59	III	alive	843	23.1	9.9	38.6	0.1	0	24.1	7.3	0.2
DO46396	64	III	dead	583	33.4	7.8	31.2	0	0.1	19.3	6.9	0
DO46448	45	III	dead	654	43.3	7.9	241.9	0.1	0	27.8	6.8	0.4
DO46588	74	III	dead	438	18.3	14.8	74.5	0.5	0.4	38.5	6.7	0.2
DO46352	60	III	dead	677	11.7	5.8	19.4	0	0	8.9	6.5	0
DO46358	60	III	dead	423	27.6	14.4	169.8	0.1	0	21.8	6.4	1.1
DO46463	49	III	dead	2451	89.5	11	75.8	0.1	0	21.2	6	0.2
DO46458	67	IV	alive	3787	30	13.3	55.2	0	0	40.2	5.8	0.5
DO46338	59	III	dead	596	57.7	10.4	129.9	0.4	6.9	23.2	5.6	0.1
DO46432	56	III	alive	2218	21.7	7.6	39.9	0	0	15.5	5.6	0.4
DO46350	66	III	dead	886	24.7	7.4	42.6	0.1	0	8.2	5.3	0.3
DO46384	55	III	dead	614	36.5	8.4	40.3	0	0	31.5	5.1	1.9
DO46374	69	III	dead	1409	20	9	88.9	0.1	0	17	4.9	0.5
DO46394	61	IV	dead	1039	45.2	10.3	62.5	0.2	0	11.9	3.8	0.3
DO46392	48	III	dead	628	42.6	8.4	103.1	0.1	0	14.7	3.3	0.4
DO46602	74	III	dead	205	31.6	25.5	33.3	0.2	0	19	3	0.3
DO46331	72	III	dead	612	26.6	3.7	4.8	0	0	7.7	2.6	0.4
DO46398	66	III	alive	437	43.5	11.4	118.6	0.7	0	28.3	2.5	0.3
DO46402	77	III	dead	511	52.3	10.7	135	0.1	0.1	29.6	1	0.2

Note: Data entries listed on the Human Protein Atlas version 24.0 as of 2 May 2025.

Table S6: Data corresponding to human ovarian tumor (n=85) and non-tumor samples (n=23) from the Clinical Proteomic Tumor Analysis Consortium (CPTAC) cohort (n=108) (PDC000110) (129, 130) that were retrieved from the Human Protein Atlas (127). Information per sample includes sample ID, sample type, and relative quantification of select proteins.

Sample ID	Sample Type	HSPG2 (nRPX)	KMT2C (nRPX)	MMP14 (nRPX)	NCOR2 (nRPX)	SPTBN2 (nRPX)
05387aad-2a1f-42c2-8d6f-950598_D3	Tumor	0.4	-0.6	-0.1	-0.1	0.1
0745488e-0bfb-4d1f-8228-dedf76_D1_D2	Normal	0.5	0.9	0	0.3	-0.2
08296da7-70ea-44df-8646-09f2cc_D2	Tumor	0.7	-0.1	0.7	0.1	-0.9
092af714-b97a-457c-b0e9-8c20e8_D2	Normal	0.8	0.4	-0.2	0.2	-0.1
0c87932d-b6fd-4eb5-b86a-a34d07_D2	Tumor	-0.1	0.4	-1.4	-0.1	0.6
0cc794c3-5bf5-4621-b238-58a0f6_D2	Tumor	-0.1	0.8	-1	0.2	0.2
10c2d629-0287-4511-ac25-f29031_D2	Tumor	0.1	-0.1	-0.7	0.2	-0.1
15524912-685a-47ce-a824-567848_D2	Tumor	0.5	0	-0.4	0.7	-0.5
1556af7e-c2ae-4fce-9aae-87c459_D1_D2	Normal	0.5	0	0	0.3	-0.2
161317f3-1a39-438e-af45-cd2502_D2	Tumor	-0.1	0.5	-0.9	0.4	-0.7
1772e913-f62e-446a-8cf3-720348_D2	Tumor	0	0.1	-0.2	0.4	-0.1
1a10c200-3895-4648-b951-94f99f_D2	Tumor	0	0.4	-0.3	0.8	-0.8
1abbcb28-f221-4cd4-8c01-37b9bb_D1_D2	Normal	1.2	0.2	-0.5	0.5	0
1b2ab076-c72f-4bbe-b9f9-657d24_D2	Tumor	0.6	0.5	0	0.7	-0.7
1c9145ad-1981-4871-aa7e-8b11de_D2	Tumor	0.2	0	-0.7	0	-0.4
1db5574d-aca7-403c-8100-6e705f_D2	Normal	1	-0.4	-0.4	0.4	-0.7
1de8d3d8-fc0e-4be0-8aa7-127c7b_D2	Tumor	0.3	0.1	-0.3	0	-0.3
2115808b-6a0d-49bc-a8d7-b83bde_D2	Tumor	0.9	0.3	0.1	0.5	-1.2
21efd60f-6a31-4b98-9477-16a905_D2	Normal	0.6	-0.2	-0.4	0.1	0.3
2224c03f-b7b1-4368-887e-2b4cdf_D2	Tumor	0.1	0.1	-0.6	0.4	-0.3
2433b136-fc7b-4cea-b958-bc6efb_D2	Tumor	-0.1	0.4	-0.5	0	-0.7
2563cb02-d58a-4286-8634-7502cd_D2	Tumor	0.2	0.6	-0.6	0.6	-0.6
256ac9f5-1e19-44b4-92fb-fef3fc_D2	Tumor	-0.1	0	-0.8	1.4	-0.1
27f3ca2d-a86f-479d-8e7e-5307c7_D2	Tumor	0	0.6	-0.9	0.2	-0.3
285fed94-1495-448e-879b-b55de9_D2	Tumor	0.6	0.3	-0.2	0.2	-0.2
28d0abdb-f682-4bd3-9425-a9c508_D2	Tumor	0.5	0.4	0.2	0.7	-0.3
2b4ce90c-8036-4207-ac3e-54a76f_D2	Tumor	0.5	-0.1	-0.2	0.8	-0.2
2b73e01c-347a-4dae-a71d-752aeb_D2	Normal	1.4	0.7	-0.3	0.2	-0.5
2dc28da8-7ff1-4b9b-b5bd-17cc25_D2	Tumor	-0.1	0.7	-0.7	0.4	-0.8
2ed8beb2-bc63-4bad-b5fe-1916a0_D2	Tumor	0.1	0.9	-0.8	0.1	0.2
2f901c90-617e-46da-9fac-d68369_D2	Tumor	0.1	0.7	-0.8	0.2	-0.3
318d53c3-216b-4de1-a098-77d6dd_D2	Tumor	0.2	0.4	-0.4	0.5	-0.2
31c15e30-21d4-4843-bd4a-9cd0d8_D2	Normal	0.5	0.7	-0.7	0.4	0.2
340a3769-3031-41ae-bb08-a2f696_D2	Normal	1	0.2	-0.3	0.2	-0.4
347e379b-77c0-4dd3-aa25-18c9d1_D2	Tumor	0.3	0.6	-0.6	0.7	-0.4
3490835c-c004-45ed-91f3-7c206f_D2	Tumor	0.7	0.3	-0.3	0.6	-0.2
35a345a4-e954-4c81-ae3f-7797cd_D2	Tumor	0	-0.4	-0.1	-0.3	0.2
3946a96c-e0ae-442f-83b4-a9350e_D2	Tumor	0.4	0.1	-0.5	0.4	-0.6
3a380577-c134-47ba-a1ce-988d7c_D2	Tumor	-0.2	0.4	-0.6	0.6	-0.5
3d3e1888-d67e-4c69-ba83-112d0f_D2	Tumor	0.6	-0.1	0.2	0.6	-0.4
3dcf8b3c-c0c5-4dfa-9543-5d30fb_D2	Tumor	0	0.7	0.3	0.4	-0.9
3e746bea-ed68-40e6-b348-6ddf08_D2	Normal	0.9	0.8	-0.3	-0.1	0.5
3fbf076a-824b-4eac-b6dc-bf5e59_D2	Normal	0.4	0.2	-0.4	0.3	0.3
41d0619e-b299-42e2-ac32-29e784_D2	Tumor	0.7	0.3	-0.1	0.4	-1.1
42d860a3-8a0c-4c44-a09a-9ad8cf_D2	Tumor	-0.1	0.8	0.2	0.3	0.8
46c73531-f531-4ec3-97fe-b39d9d_D2	Tumor	0.4	1	0.2	0.2	-0.4
49bd51fe-48a1-4828-b66e-b30c38_D2	Tumor	-0.3	0.6	-0.3	0.5	-0.8
4ccb249a-6722-491e-97e9-e3b4e7_D2	Tumor	0.4	0.3	0.4	0.5	-0.3
50997c44-1c8b-4d34-b535-4a6cc2_D2	Tumor	0	-0.4	-0.7	0.5	-0.5
51a4ce2f-4166-4c6b-a7e0-942d0a_D2	Tumor	0.1	0.2	-0.4	-0.1	-0.6
51c1c79e-5a8e-4b0a-bf92-1c0cab_D2	Tumor	-0.3	0.8	0.4	0.2	0.4
52c064a7-78d9-4a1a-85b4-27c6d1_D2	Tumor	-0.3	0.5	-0.2	0.1	-0.2
558333b0-ea23-4303-9e3c-4932b1_D2	Tumor	0	0.9	-0.6	0.6	-0.2
5c3971ee-87c7-4dff-b66e-f18892_D2	Tumor	0.1	-0.2	-1	0.4	-0.5
5ea22f5e-307b-491d-801f-8b7fa6_D2	Tumor	0	0.6	-0.5	0.5	-0.2
61f76fe6-a1c6-41b8-bdc2-2f9209_D2	Normal	1.6	0.3	-0.7	0.1	0
627e6e36-3dca-4ee5-9755-6f3510_D2	Tumor	-0.3	0.1	-0.3	0.3	-0.3

62f5840e-6cd7-4200-b651-645e53_D2	Tumor	0.5	0.3	-0.3	0.4	-0.3
6319b36b-533c-4e86-8e12-80552e_D3	Tumor	1.1	0.2	0.4	0.1	-0.5
6530147a-efe9-4df6-ac2f-03ece3_D2	Tumor	1.3	0	0.1	0.4	-0.6
685ec3c2-e67a-40d3-9eb3-aa01c9_D1_D2	Normal	1	0.5	0.1	-0.1	-0.4
69d552be-7cb2-4bb4-b602-a27b3e_D2	Tumor	0.6	0.4	0.7	0.3	-0.2
6e209757-a7f4-45af-b5f3-2ebc02_D2	Normal	1.1	0	-0.2	0.3	0.1
7069e990-4a4e-4984-a66d-4d835a_D2	Tumor	0.1	0.3	-0.7	0.3	-0.3
74a45e5a-a35a-4c60-a4b4-f3f5ea_D2	Tumor	-0.5	-0.3	-0.5	-0.3	-0.2
76e77c6b-c80e-4391-bdbb-e1dcea_D2	Tumor	0.4	0.1	-0.7	0.4	-0.5
79b60f32-6cca-4cb6-9a1e-22a3ac_D2	Tumor	0.3	0.2	-0.5	0.4	-0.7
7c7ce2d7-6c8a-424e-8e6c-d14ad1_D2	Tumor	0.9	0.6	-0.1	0.3	-0.3
7d953d1d-84b6-4ecd-b1d7-1ed865_D2	Tumor	0.3	0.4	-0.1	0.6	-0.5
7edfd606-4bee-4529-9fec-1ca7f0_D2	Normal	0.9	0.5	-1.3	0.2	-0.1
80873a0c-1f40-4d51-bab1-3f2071_D2	Tumor	0.5	-0.2	-0.4	0.3	-0.3
8234f8ea-bdbd-45b0-bd0b-16f1e7_D2	Tumor	0.2	0.4	-0.4	0.1	-0.5
8d78194e-fbef-4819-a7b5-b73ae9_D2	Tumor	-0.3	0.7	-0.5	0	-0.8
8e17b45e-6a06-4e4e-913d-7b1edb_D2	Tumor	-0.4	0.8	-1	0.2	0
8ed739dd-ae0f-4881-971a-db3cf4_D1_D2	Normal	0.9	0.7	-1.2	-0.3	0.4
91b287fd-eadb-442d-abcd-20b741_D2	Tumor	0.3	0.6	-0.2	0.7	-0.5
927f0958-db5a-4bc5-92b7-2a3695_D2	Tumor	0.6	-0.4	-0.3	0	-0.3
994f9b8e-46cb-413f-8ec8-2c2fed_D2	Normal	1	0.2	-0.2	0.2	0
9a7a86d3-bbf2-4d36-941d-c75c03_D1_D2	Tumor	0.9	0.5	0	0.2	-0.2
9b90eb78-2f50-4aeb-b010-d642f9_D2	Tumor	0.7	0.3	-0.8	0.5	-0.6
9c8e78bf-b03e-4ba0-9909-5ff070_D2	Tumor	-0.3	0.1	-0.6	0.2	-0.4
9e0d47e0-f633-468b-b82b-4d752d_D2	Tumor	-0.1	0.3	-1.2	0.1	0.2
a03d1103-b809-42c4-ad5c-5aff58_D2	Tumor	-0.1	1.1	-0.9	1.3	-0.3
a3a26070-fb84-406a-8280-d555e6_D2	Normal	0.6	0.4	0.2	-0.4	0
a583d3ff-8165-43e9-b764-23b7ef_D1_D2	Normal	1.2	0.7	-0.4	0.2	-0.1
a65d8e76-640f-4341-a561-afbb57_D2	Normal	0.9	-0.9	-0.3	0.2	-0.6
a8853382-b726-4ce8-98d5-ca89df_D2	Tumor	-0.5	0.1	-0.1	0.6	-0.4
af95e2c6-25c4-4ab8-bf06-3e15ff_D2	Tumor	-0.2	0.3	-0.6	0.3	-0.5
b22659b9-e12c-4725-a7db-a833de_D1_D2	Normal	0.4	-0.1	-0.3	0.4	0
b9c1f22e-61f7-45ed-b3b4-5094c7_D2	Normal	0.9	-0.1	0.2	0	-0.4
bd266bdd-12e4-42cb-9c49-911bfc_D2	Tumor	0.6	0	0	0.4	-0.4
c17b0ff9-9057-4848-a0cc-abb25f_D2	Tumor	0	0.3	-0.6	-0.2	-0.3
ce1e5dfc-2ef1-4633-af93-5ea4d9_D2	Tumor	0.3	0.8	0.1	0.5	-0.5
cfd76f9a-ce60-4ec7-8e6b-a8fc31_D3	Tumor	0.6	0.3	-0.3	0	0
d109a3c0-350e-47f5-bda0-61899c_D2	Tumor	0.4	0	0.3	0.8	-0.7
d38f40fb-dd78-43b2-8b7b-4b6977_D2	Tumor	0.7	0.4	-0.7	0.9	-0.4
d4791079-c826-4fc7-9d9b-ee20ba_D2	Tumor	0.2	0	0.1	0.3	0.1
e23eb014-69c1-460a-b9d4-9786c8_D2	Normal	1.1	0.1	-0.6	-0.1	0.1
e3ebe010-cd31-4b5d-8fcb-b7103b_D2	Tumor	-0.5	0.2	-0.1	-0.1	0.1
e6850782-fd2a-4c53-b682-1852fe_D2	Tumor	0	-0.4	-0.4	-0.1	0
e7b656bf-3c34-4ba1-bdf3-c7eefe_D2	Tumor	-0.3	0.1	-0.1	0.4	-0.2
e9d416e7-f661-400a-896e-388545_D2	Tumor	0.2	0.3	-0.4	0.4	0
eb0a53df-7620-4024-8d2d-051142_D2	Tumor	0.8	0	-0.2	0.5	-1.1
eb3f3494-7057-4afe-b212-e6e9a3_D2	Tumor	-0.4	-0.4	-0.8	0.1	-0.1
efc971f8-54c5-4531-a792-705ecf_D2	Tumor	0.3	0.8	-0.1	0.3	-0.4
f6c215ea-5381-4521-bb61-be8e97_D2	Tumor	0.7	0.1	-0.7	0.6	-0.4
fbb41c94-aabd-49cc-90b2-882c3e_D2	Tumor	-0.5	0	-0.1	0.2	-0.1
fe83e13f-9754-403b-8f74-d6caf8_D2	Tumor	0.6	0.1	-0.3	0.4	-0.6
Note: Data entries listed on the Human Protein Atlas version 24.0 as of 2 May 2025.						

Table S7: Structural analysis of available MMP14 structures containing amino acid residues 238-244.

MMP14 Structure (PDB ID or AlphaFold Protein Structure Database ID)	Chain	Solvent-Accessible Surface Area of $MMP14_{238\text{-}244}$ (VHELGHA) (Å^2)
1BQQ	M	893.93
1BUV	M	893.93
3MA2	A	883.53
3MA2	D	880.2
5H0U	A	875.67
AF-P50281-F1-model_v4	A	889.72

Table S8: Comparisons between available MMP14 structures containing amino acid residues 238-244 and predicted structures of the CVPELGHEC nonapeptide (PEP-FOLD2, AlphaFold2, or AlphaFold3).

MMP14 Structure (PDB ID or AlphaFold Protein Structure Database ID)	Chain	MMP14$_{238\text{-}244}$ vs. PF2 Peptide			MMP14$_{238\text{-}244}$ vs. AF2 Peptide			MMP14$_{238\text{-}244}$ vs. AF3 Peptide		
		# Pruned Cα Pairs	Pruned RMSD (Å)	RMSD of All 7 Cα Pairs (Å)	# Pruned Cα Pairs	Pruned RMSD (Å)	RMSD of All 7 Cα Pairs (Å)	# Pruned Cα Pairs	Pruned RMSD (Å)	RMSD of All 7 Cα Pairs (Å)
1BQQ	M	5	0.452	4.623	4	1.25	4.074	5	0.535	5.202
1BUV	M	5	0.452	4.623	4	1.25	4.074	5	0.535	5.202
3MA2	A	5	0.524	4.457	4	1.197	4.036	5	0.617	5.046
3MA2	D	5	0.525	4.412	4	1.22	4.014	5	0.608	5.029
5H0U	A	5	0.465	4.508	3	0.184	4.395	5	0.536	5.070
AF-P50281-F1-model_v4	A	5	0.48	4.516	4	1.27	4.066	5	0.561	5.105

Dataset S1 (separate file): Top-scoring proteins of the human proteome according to BLASTP bit-score that the consensus motif (VPELGHE) maps to and their corresponding amino acid sequence alignments.

Supplementary References

1. Kramer, A., Green, J., Pollard, J., Jr., and Tugendreich, S. (2014) Causal analysis approaches in Ingenuity Pathway Analysis. *Bioinformatics* **30**, 523-530
2. Goddard, T. D., Huang, C. C., Meng, E. C., Pettersen, E. F., Couch, G. S., Morris, J. H., and Ferrin, T. E. (2018) UCSF ChimeraX: meeting modern challenges in visualization and analysis. *Protein Sci* **27**, 14-25
3. Fernandez-Catalan, C., Bode, W., Huber, R., Turk, D., Calvete, J. J., Lichte, A., Tschesche, H., and Maskos, K. (1998) Crystal structure of the complex formed by the membrane type 1-matrix metalloproteinase with the tissue inhibitor of metalloproteinases-2, the soluble progelatinase A receptor. *EMBO J* **17**, 5238-5248
4. Grossman, M., Tworowski, D., Dym, O., Lee, M. H., Levy, Y., Murphy, G., and Sagi, I. (2010) The intrinsic protein flexibility of endogenous protease inhibitor TIMP-1 controls its binding interface and affects its function. *Biochemistry* **49**, 6184-6192
5. Decaneto, E., Vasilevskaya, T., Kutin, Y., Ogata, H., Grossman, M., Sagi, I., Havenith, M., Lubitz, W., Thiel, W., and Cox, N. (2017) Solvent water interactions within the active site of the membrane type I matrix metalloproteinase. *Phys Chem Chem Phys* **19**, 30316-30331
6. Jumper, J., Evans, R., Pritzel, A., Green, T., Figurnov, M., Ronneberger, O., Tunyasuvunakool, K., Bates, R., Zidek, A., Potapenko, A., Bridgland, A., Meyer, C., Kohl, S. A. A., Ballard, A. J., Cowie, A., Romera-Paredes, B., Nikolov, S., Jain, R., Adler, J., Back, T., Petersen, S., Reiman, D., Clancy, E., Zielinski, M., Steinegger, M., Pacholska, M., Berghammer, T., Bodenstein, S., Silver, D., Vinyals, O., Senior, A. W., Kavukcuoglu, K., Kohli, P., and Hassabis, D. (2021) Highly accurate protein structure prediction with AlphaFold. *Nature* **596**, 583-589
7. Varadi, M., Anyango, S., Deshpande, M., Nair, S., Natassia, C., Yordanova, G., Yuan, D., Stroe, O., Wood, G., Laydon, A., Zidek, A., Green, T., Tunyasuvunakool, K., Petersen, S., Jumper, J., Clancy, E., Green, R., Vora, A., Lutfi, M., Figurnov, M., Cowie, A., Hobbs, N., Kohli, P., Kleywegt, G., Birney, E.,

Hassabis, D., and Velankar, S. (2022) AlphaFold Protein Structure Database: massively expanding the structural coverage of protein-sequence space with high-accuracy models. *Nucleic Acids Res* **50**, D439-D444

8. UniProt Consortium. (2025) UniProt: the Universal Protein Knowledgebase in 2025. *Nucleic Acids Res* **53**, D609-D617
9. Shen, Y., Maupetit, J., Derreumaux, P., and Tuffery, P. (2014) Improved PEP-FOLD approach for peptide and miniprotein structure prediction. *J Chem Theory Comput* **10**, 4745-4758
10. Abramson, J., Adler, J., Dunger, J., Evans, R., Green, T., Pritzel, A., Ronneberger, O., Willmore, L., Ballard, A. J., Bambrick, J., Bodenstein, S. W., Evans, D. A., Hung, C. C., O'Neill, M., Reiman, D., Tunyasuvunakool, K., Wu, Z., Zemgulyte, A., Arvaniti, E., Beattie, C., Bertolli, O., Bridgland, A., Cherepanov, A., Congreve, M., Cowen-Rivers, A. I., Cowie, A., Figurnov, M., Fuchs, F. B., Gladman, H., Jain, R., Khan, Y. A., Low, C. M. R., Perlin, K., Potapenko, A., Savy, P., Singh, S., Stecula, A., Thillaisundaram, A., Tong, C., Yakneen, S., Zhong, E. D., Zielinski, M., Zidek, A., Bapst, V., Kohli, P., Jaderberg, M., Hassabis, D., and Jumper, J. M. (2024) Accurate structure prediction of biomolecular interactions with AlphaFold 3. *Nature* **630**, 493-500
11. Mirdita, M., Schutze, K., Moriwaki, Y., Heo, L., Ovchinnikov, S., and Steinegger, M. (2022) ColabFold: making protein folding accessible to all. *Nat Methods* **19**, 679-682
12. Lamiable, A., Thevenet, P., Rey, J., Vavrusa, M., Derreumaux, P., and Tuffery, P. (2016) PEP-FOLD3: faster de novo structure prediction for linear peptides in solution and in complex. *Nucleic Acids Res* **44**, W449-454
13. Vidal, C. I., Mintz, P. J., Lu, K., Ellis, L. M., Manenti, L., Giavazzi, R., Gershenson, D. M., Broaddus, R., Liu, J., Arap, W., and Pasqualini, R. (2004) An HSP90-mimic peptide revealed by fingerprinting the pool of antibodies from ovarian cancer patients. *Oncogene* **23**, 8859-8867
14. Liu, Y., Yasukawa, M., Chen, K., Hu, L., Broaddus, R. R., Ding, L., Mardis, E. R., Spellman, P., Levine, D. A., Mills, G. B., Shmulevich, I., Sood, A. K., and Zhang, W. (2015) Association of somatic mutations of ADAMTS genes With

chemotherapy sensitivity and survival in high-grade serous ovarian carcinoma. *JAMA Oncol* **1**, 486-494

15. Lima, M. A., Dos Santos, L., Turri, J. A., Nonogaki, S., Buim, M., Lima, J. F., de Jesus Viana Pinheiro, J., Bueno de Toledo Osorio, C. A., Soares, F. A., and Freitas, V. M. (2016) Prognostic value of ADAMTS proteases and their substrates in epithelial ovarian cancer. *Pathobiology* **83**, 316-326
16. Yasukawa, M., Liu, Y., Hu, L., Cogdell, D., Gharpure, K. M., Pradeep, S., Nagaraja, A. S., Sood, A. K., and Zhang, W. (2017) ADAMTS16 mutations sensitize ovarian cancer cells to platinum-based chemotherapy. *Oncotarget* **8**, 88410-88420
17. Long, X., Liu, L., Zhao, Q., Xu, X., Liu, P., Zhang, G., and Lin, J. (2022) Comprehensive analysis of tripterine anti-ovarian cancer effects using weighted gene co-expression network analysis and molecular docking. *Med Sci Monit* **28**, e932139
18. Stawerski, P., Wagrowska-Danilewicz, M., Stasikowska, O., and Danilewicz, M. (2010) Immunoexpression of CAS protein is augmented in high grade serous ovarian tumors. *Pol J Pathol* **61**, 219-223
19. Nick, A. M., Stone, R. L., Armaiz-Pena, G., Ozpolat, B., Tekedereli, I., Graybill, W. S., Landen, C. N., Villares, G., Vivas-Mejia, P., Bottsford-Miller, J., Kim, H. S., Lee, J. S., Kim, S. M., Baggerly, K. A., Ram, P. T., Deavers, M. T., Coleman, R. L., Lopez-Berestein, G., and Sood, A. K. (2011) Silencing of p130cas in ovarian carcinoma: a novel mechanism for tumor cell death. *J Natl Cancer Inst* **103**, 1596-1612
20. Horwitz, V., Davidson, B., Stern, D., Trope, C. G., Tavor Re'em, T., and Reich, R. (2016) Ezrin is associated with disease progression in ovarian carcinoma. *PLoS One* **11**, e0162502
21. Wen, Y., Chelariu-Raicu, A., Umamaheswaran, S., Nick, A. M., Stur, E., Hanjra, P., Jiang, D., Jennings, N. B., Chen, X., Corvigno, S., Glassman, D., Lopez-Berestein, G., Liu, J., Hung, M. C., and Sood, A. K. (2022) Endothelial p130cas confers resistance to anti-angiogenesis therapy. *Cell Rep* **38**, 110301

22. Ma, Y., Ma, L., Guo, Q., and Zhang, S. (2010) Expression of bone morphogenetic protein-2 and its receptors in epithelial ovarian cancer and their influence on the prognosis of ovarian cancer patients. *J Exp Clin Cancer Res* **29**, 85

23. Oseto, K., Suzumori, N., Nishikawa, R., Nishikawa, H., Arakawa, A., Ozaki, Y., Asai, H., Kawai, M., Mizuno, K., Takahashi, S., Shirai, T., Yamada-Namikawa, C., Nakanishi, M., Kajiyama, H., Kikkawa, F., and Sugiura-Ogasawara, M. (2014) Mutational analysis of FOXL2 p.C134W and expression of bone morphogenetic protein 2 in Japanese patients with granulosa cell tumor of ovary. *J Obstet Gynaecol Res* **40**, 1197-1204

24. Choi, Y. J., Ingram, P. N., Yang, K., Coffman, L., Iyengar, M., Bai, S., Thomas, D. G., Yoon, E., and Buckanovich, R. J. (2015) Identifying an ovarian cancer cell hierarchy regulated by bone morphogenetic protein 2. *Proc Natl Acad Sci U S A* **112**, E6882-6888

25. Fukuda, T., Fukuda, R., Tanabe, R., Koinuma, D., Koyama, H., Hashizume, Y., Moustakas, A., Miyazono, K., and Heldin, C. H. (2020) BMP signaling is a therapeutic target in ovarian cancer. *Cell Death Discov* **6**, 139

26. Fukuda, T., Fukuda, R., Miyazono, K., and Heldin, C. H. (2021) Tumor promoting effect of BMP signaling in endometrial cancer. *Int J Mol Sci* **22**

27. Cai, X., Wang, J., and Xin, X. (2012) CIAPIN1 nuclear accumulation predicts poor clinical outcome in epithelial ovarian cancer. *World J Surg Oncol* **10**, 112

28. Nymoen, D. A., Holth, A., Hetland Falkenthal, T. E., Trope, C. G., and Davidson, B. (2015) CIAPIN1 and ABCA13 are markers of poor survival in metastatic ovarian serous carcinoma. *Mol Cancer* **14**, 44

29. Zheng, W., Luo, M. P., Welt, C., Lambert-Messerlian, G., Sung, C. J., Zhang, Z., Ying, S. Y., Schneyer, A. L., Lauchlan, S. C., and Felix, J. C. (1998) Imbalanced expression of inhibin and activin subunits in primary epithelial ovarian cancer. *Gynecol Oncol* **69**, 23-31

30. Choi, Y. L., Kim, H. S., and Ahn, G. (2000) Immunoexpression of inhibin alpha subunit, inhibin/activin betaA subunit and CD99 in ovarian tumors. *Arch Pathol Lab Med* **124**, 563-569

31. Zheng, W., Lu, J. J., Luo, F., Hsieh, J., Wang, C. Y., Zhang, C., Chang, L., Cho, M. M., and Stanczyk, F. Z. (2000) Tumor stroma as the main source of inhibin production in ovarian epithelial tumors. *Am J Reprod Immunol* **44**, 104-113
32. Choi, K. C., Kang, S. K., Nathwani, P. S., Cheng, K. W., Auersperg, N., and Leung, P. C. (2001) Differential expression of activin/inhibin subunit and activin receptor mRNAs in normal and neoplastic ovarian surface epithelium (OSE). *Mol Cell Endocrinol* **174**, 99-110
33. Cobellis, L., Cataldi, P., Reis, F. M., De Palo, G., Raspagliesi, F., Pilotti, S., Arcuri, F., and Petraglia, F. (2001) Gonadal malignant germ cell tumors express immunoreactive inhibin/activin subunits. *Eur J Endocrinol* **145**, 779-784
34. Zhu, Q., Yang, X., and Lv, Y. (2024) HERC4 modulates ovarian cancer cell proliferation by regulating SMO-elicited hedgehog signaling. *Biochim Biophys Acta Gen Subj* **1868**, 130557
35. Li, Y., Li, H., Yang, B., Wei, J., Zhen, C., and Feng, L. (2020) Clinical significance of PI3 and HLA-DOB as potential prognostic predicators for ovarian cancer. *Transl Cancer Res* **9**, 466-476
36. Lai, J., Chien, J., Staub, J., Avula, R., Greene, E. L., Matthews, T. A., Smith, D. I., Kaufmann, S. H., Roberts, L. R., and Shridhar, V. (2003) Loss of HSulf-1 up-regulates heparin-binding growth factor signaling in cancer. *J Biol Chem* **278**, 23107-23117
37. Davies, E. J., Blackhall, F. H., Shanks, J. H., David, G., McGown, A. T., Swindell, R., Slade, R. J., Martin-Hirsch, P., Gallagher, J. T., and Jayson, G. C. (2004) Distribution and clinical significance of heparan sulfate proteoglycans in ovarian cancer. *Clin Cancer Res* **10**, 5178-5186
38. Kanchi, K. L., Johnson, K. J., Lu, C., McLellan, M. D., Leiserson, M. D., Wendl, M. C., Zhang, Q., Koboldt, D. C., Xie, M., Kandoth, C., McMichael, J. F., Wyczalkowski, M. A., Larson, D. E., Schmidt, H. K., Miller, C. A., Fulton, R. S., Spellman, P. T., Mardis, E. R., Druley, T. E., Graubert, T. A., Goodfellow, P. J., Raphael, B. J., Wilson, R. K., and Ding, L. (2014) Integrated analysis of germline and somatic variants in ovarian cancer. *Nat Commun* **5**, 3156

39. Li, J., Chen, Z., Xiao, W., Liang, H., Liu, Y., Hao, W., Zhang, Y., and Wei, F. (2023) Chromosome instability region analysis and identification of the driver genes of the epithelial ovarian cancer cell lines A2780 and SKOV3. *J Cell Mol Med* **27**, 3259-3270
40. Afzal, S., Lalani, E. N., Poulsom, R., Stubbs, A., Rowlinson, G., Sato, H., Seiki, M., and Stamp, G. W. (1998) MT1-MMP and MMP-2 mRNA expression in human ovarian tumors: possible implications for the role of desmoplastic fibroblasts. *Hum Pathol* **29**, 155-165
41. Sakata, K., Shigemasa, K., Nagai, N., and Ohama, K. (2000) Expression of matrix metalloproteinases (MMP-2, MMP-9, MT1-MMP) and their inhibitors (TIMP-1, TIMP-2) in common epithelial tumors of the ovary. *Int J Oncol* **17**, 673-681
42. Davidson, B., Goldberg, I., Gotlieb, W. H., Kopolovic, J., Ben-Baruch, G., Nesland, J. M., Berner, A., Bryne, M., and Reich, R. (1999) High levels of MMP-2, MMP-9, MT1-MMP and TIMP-2 mRNA correlate with poor survival in ovarian carcinoma. *Clin Exp Metastasis* **17**, 799-808
43. Davidson, B., Reich, R., Goldberg, I., Gotlieb, W. H., Kopolovic, J., Berner, A., Ben-Baruch, G., Bryne, M., and Nesland, J. M. (2001) Ets-1 messenger RNA expression is a novel marker of poor survival in ovarian carcinoma. *Clin Cancer Res* **7**, 551-557
44. Drew, A. F., Blick, T. J., Lafleur, M. A., Tim, E. L., Robbie, M. J., Rice, G. E., Quinn, M. A., and Thompson, E. W. (2004) Correlation of tumor- and stromal-derived MT1-MMP expression with progression of human ovarian tumors in SCID mice. *Gynecol Oncol* **95**, 437-448
45. Barbolina, M. V., Adley, B. P., Ariztia, E. V., Liu, Y., and Stack, M. S. (2007) Microenvironmental regulation of membrane type 1 matrix metalloproteinase activity in ovarian carcinoma cells via collagen-induced EGR1 expression. *J Biol Chem* **282**, 4924-4931
46. Cowden Dahl, K. D., Zeineldin, R., and Hudson, L. G. (2007) PEA3 is necessary for optimal epidermal growth factor receptor-stimulated matrix metalloproteinase expression and invasion of ovarian tumor cells. *Mol Cancer Res* **5**, 413-421

47. Sodek, K. L., Ringuette, M. J., and Brown, T. J. (2007) MT1-MMP is the critical determinant of matrix degradation and invasion by ovarian cancer cells. *Br J Cancer* **97**, 358-367

48. Liao, X., Siu, M. K., Au, C. W., Wong, E. S., Chan, H. Y., Ip, P. P., Ngan, H. Y., and Cheung, A. N. (2009) Aberrant activation of hedgehog signaling pathway in ovarian cancers: effect on prognosis, cell invasion and differentiation. *Carcinogenesis* **30**, 131-140

49. Moss, N. M., Liu, Y., Johnson, J. J., Debiase, P., Jones, J., Hudson, L. G., Munshi, H. G., and Stack, M. S. (2009) Epidermal growth factor receptor-mediated membrane type 1 matrix metalloproteinase endocytosis regulates the transition between invasive versus expansive growth of ovarian carcinoma cells in three-dimensional collagen. *Mol Cancer Res* **7**, 809-820

50. Moss, N. M., Barbolina, M. V., Liu, Y., Sun, L., Munshi, H. G., and Stack, M. S. (2009) Ovarian cancer cell detachment and multicellular aggregate formation are regulated by membrane type 1 matrix metalloproteinase: a potential role in I.p. metastatic dissemination. *Cancer Res* **69**, 7121-7129

51. Koshikawa, N., Mizushima, H., Minegishi, T., Eguchi, F., Yotsumoto, F., Nabeshima, K., Miyamoto, S., Mekada, E., and Seiki, M. (2011) Proteolytic activation of heparin-binding EGF-like growth factor by membrane-type matrix metalloproteinase-1 in ovarian carcinoma cells. *Cancer Sci* **102**, 111-116

52. Jiang, L., Siu, M. K., Wong, O. G., Tam, K. F., Lam, E. W., Ngan, H. Y., Le, X. F., Wong, E. S., Chan, H. Y., and Cheung, A. N. (2010) Overexpression of proto-oncogene FBI-1 activates membrane type 1-matrix metalloproteinase in association with adverse outcome in ovarian cancers. *Mol Cancer* **9**, 318

53. Ling Poon, S., Lau, M. T., Hammond, G. L., and Leung, P. C. (2011) Gonadotropin-releasing hormone-II increases membrane type I metalloproteinase production via beta-catenin signaling in ovarian cancer cells. *Endocrinology* **152**, 764-772

54. Brun, J. L., Cortez, A., Lesieur, B., Uzan, S., Rouzier, R., and Darai, E. (2012) Expression of MMP-2, -7, -9, MT1-MMP and TIMP-1 and -2 has no prognostic

relevance in patients with advanced epithelial ovarian cancer. *Oncol Rep* **27**, 1049-1057

55. Kaimal, R., Aljumaily, R., Tressel, S. L., Pradhan, R. V., Covic, L., Kuliopulos, A., Zarwan, C., Kim, Y. B., Sharifi, S., and Agarwal, A. (2013) Selective blockade of matrix metalloprotease-14 with a monoclonal antibody abrogates invasion, angiogenesis, and tumor growth in ovarian cancer. *Cancer Res* **73**, 2457-2467
56. Nakayama, I., Shibazaki, M., Yashima-Abo, A., Miura, F., Sugiyama, T., Masuda, T., and Maesawa, C. (2013) Loss of HOXD10 expression induced by upregulation of miR-10b accelerates the migration and invasion activities of ovarian cancer cells. *Int J Oncol* **43**, 63-71
57. Bruney, L., Conley, K. C., Moss, N. M., Liu, Y., and Stack, M. S. (2014) Membrane-type I matrix metalloproteinase-dependent ectodomain shedding of mucin16/ CA-125 on ovarian cancer cells modulates adhesion and invasion of peritoneal mesothelium. *Biol Chem* **395**, 1221-1231
58. Vos, M. C., van der Wurff, A. A., Bulten, J., Kruitwagen, R., Feijen, H., van Kuppevelt, T. H., Hendriks, T., and Massuger, L. F. (2016) Limited independent prognostic value of MMP-14 and MMP-2 expression in ovarian cancer. *Diagn Pathol* **11**, 34
59. Vos, M. C., Hollemans, E., Ezendam, N., Feijen, H., Boll, D., Pijlman, B., van der Putten, H., Klinkhamer, P., van Kuppevelt, T. H., van der Wurff, A. A., and Massuger, L. F. (2016) MMP-14 and CD44 in epithelial-to-mesenchymal transition (EMT) in ovarian cancer. *J Ovarian Res* **9**, 53
60. Yang, J., Kasberg, W. C., Celo, A., Liang, Z., Quispe, K., and Stack, M. S. (2017) Post-translational modification of the membrane type 1 matrix metalloproteinase (MT1-MMP) cytoplasmic tail impacts ovarian cancer multicellular aggregate dynamics. *J Biol Chem* **292**, 13111-13121
61. Takahashi, Y., Hamasaki, M., Aoki, M., Koga, K., Koshikawa, N., Miyamoto, S., and Nabeshima, K. (2018) Activated EphA2 processing by MT1-MMP is involved in malignant transformation of ovarian tumours in vivo. *Anticancer Res* **38**, 4257-4266

62. Chellini, L., Caprara, V., Spadaro, F., Sestito, R., Bagnato, A., and Rosano, L. (2019) Regulation of extracellular matrix degradation and metastatic spread by IQGAP1 through endothelin-1 receptor signalling in ovarian cancer. *Matrix Biol* **81**, 17-33
63. Vos, M. C., Hollemans, E., van der Steen, S., van Kuppevelt, T. H., van der Wurff, A. A. M., and Massuger, L. (2020) Primary ovarian tumors with lymphogenic and hematogenic metastasis express high MMP-14, which colocalizes with highly sulfated chondroitin sulfate in the stroma. *Int J Gynecol Pathol* **39**, 184-192
64. Vos, M. C., van Tilborg, A., Brands, W. J., Boll, D., van Hamont, D., van der Putten, H., Pijlman, B., van der Wurff, A. A. M., van Kuppevelt, T. H., and Massuger, L. (2019) Polymorphisms in MMP-14 and MMP-2 genes and ovarian cancer survival. *Cancer Biomark* **25**, 233-241
65. Vos, M. C., van der Wurff, A. A. M., van Kuppevelt, T. H., and Massuger, L. (2021) The role of MMP-14 in ovarian cancer: a systematic review. *J Ovarian Res* **14**, 101
66. Banville, A. C., and Nelson, B. H. (2022) Breaching B cell tolerance in the tumor microenvironment. *Cancer Cell* **40**, 356-358
67. Lin, A., Xu, H. H., Xu, D. P., Zhang, X., Wang, Q., and Yan, W. H. (2013) Multiple steps of HLA-G in ovarian carcinoma metastasis: alter NK cytotoxicity and induce matrix metalloproteinase-15 (MMP-15) expression. *Hum Immunol* **74**, 439-446
68. Wang, H., Qi, C., and Wan, D. (2021) MicroRNA-377-3p targeting MMP-16 inhibits ovarian cancer cell growth, invasion, and interstitial transition. *Ann Transl Med* **9**, 124
69. Wang, W., Liu, Y., Yang, Y., Huang, X., and Hou, Y. (2021) MMP-16 as a new biomarker for predicting prognosis and chemosensitivity of serous ovarian cancer: a study based on bioinformatics analysis. *Crit Rev Eukaryot Gene Expr* **31**, 1-8
70. Zhao, H., Yang, Z., Wang, X., Zhang, X., Wang, M., Wang, Y., Mei, Q., and Wang, Z. (2012) Triptolide inhibits ovarian cancer cell invasion by repression of matrix metalloproteinase 7 and 19 and upregulation of E-cadherin. *Exp Mol Med* **44**, 633-641

71. Wang, S., Jia, J., Liu, D., Wang, M., Wang, Z., Li, X., Wang, H., Rui, Y., Liu, Z., Guo, W., Nie, J., and Dai, H. (2019) Matrix metalloproteinase expressions play important role in prediction of ovarian cancer outcome. *Sci Rep* **9**, 11677

72. Tamada, Y., Iida, S., Aoki, D., Nozawa, S., and Irimura, T. (1999) Carbohydrate epitopes and mucins expressed by 17 human ovarian carcinoma cell lines. *Oncol Res* **11**, 233-241

73. Albarracin, C. T., Jafri, J., Montag, A. G., Hart, J., and Kuan, S. F. (2000) Differential expression of MUC2 and MUC5AC mucin genes in primary ovarian and metastatic colonic carcinoma. *Hum Pathol* **31**, 672-677

74. O'Connell, J. T., Tomlinson, J. S., Roberts, A. A., McGonigle, K. F., and Barsky, S. H. (2002) Pseudomyxoma peritonei is a disease of MUC2-expressing goblet cells. *Am J Pathol* **161**, 551-564

75. O'Connell, J. T., Hacker, C. M., and Barsky, S. H. (2002) MUC2 is a molecular marker for pseudomyxoma peritonei. *Mod Pathol* **15**, 958-972

76. Tornos, C., Soslow, R., Chen, S., Akram, M., Hummer, A. J., Abu-Rustum, N., Norton, L., and Tan, L. K. (2005) Expression of WT1, CA 125, and GCDFP-15 as useful markers in the differential diagnosis of primary ovarian carcinomas versus metastatic breast cancer to the ovary. *Am J Surg Pathol* **29**, 1482-1489

77. Ferreira, C. R., Carvalho, J. P., Soares, F. A., Siqueira, S. A., and Carvalho, F. M. (2008) Mucinous ovarian tumors associated with pseudomyxoma peritonei of adenomucinosis type: immunohistochemical evidence that they are secondary tumors. *Int J Gynecol Cancer* **18**, 59-65

78. Hirabayashi, K., Yasuda, M., Kajiwara, H., Itoh, J., Miyazawa, M., Hirasawa, T., Muramatsu, T., Murakami, M., Mikami, M., and Osamura, R. Y. (2008) Alterations in mucin expression in ovarian mucinous tumors: immunohistochemical analysis of MUC2, MUC5AC, MUC6, and CD10 expression. *Acta Histochem Cytochem* **41**, 15-21

79. Wang, J., and El-Bahrawy, M. A. (2014) Expression profile of mucins in ovarian mucinous tumors: distinguishing primary ovarian from metastatic tumors. *Int J Gynecol Pathol* **33**, 166-175

80. Wang, J., and El-Bahrawy, M. (2015) Expression profile of mucins (MUC1, MUC2, MUC5AC, and MUC6) in ovarian mucinous tumours: changes in expression from benign to malignant tumours. *Histopathology* **66**, 529-535
81. Giuntoli, R. L., 2nd, Rodriguez, G. C., Whitaker, R. S., Dodge, R., and Voynow, J. A. (1998) Mucin gene expression in ovarian cancers. *Cancer Res* **58**, 5546-5550
82. Ji, H., Isacson, C., Seidman, J. D., Kurman, R. J., and Ronnett, B. M. (2002) Cytokeratins 7 and 20, Dpc4, and MUC5AC in the distinction of metastatic mucinous carcinomas in the ovary from primary ovarian mucinous tumors: Dpc4 assists in identifying metastatic pancreatic carcinomas. *Int J Gynecol Pathol* **21**, 391-400
83. Baker, P. M., and Oliva, E. (2005) Immunohistochemistry as a tool in the differential diagnosis of ovarian tumors: an update. *Int J Gynecol Pathol* **24**, 39-55
84. Han, L., Pansare, V., Al-Abbadi, M., Husain, M., and Feng, J. (2010) Combination of MUC5ac and WT-1 immunohistochemistry is useful in distinguishing pancreatic ductal carcinoma from ovarian serous carcinoma in effusion cytology. *Diagn Cytopathol* **38**, 333-336
85. Musrap, N., Karagiannis, G. S., Saraon, P., Batruch, I., Smith, C., and Diamandis, E. P. (2014) Proteomic analysis of cancer and mesothelial cells reveals an increase in mucin 5AC during ovarian cancer and peritoneal interaction. *J Proteomics* **103**, 204-215
86. Mateoiu, C., Vitiazeva, V., Kristjansdottir, B., Weijdegard, B., Ornros, J., Gallini, R., Kamali-Moghaddam, M., Sundfeldt, K., and Karlsson, N. G. (2021) Analysis of blood group antigens on MUC5AC in mucinous ovarian cancer tissues using in situ proximity ligation assay. *Glycobiology* **31**, 1464-1471
87. Chelariu-Raicu, A., Holley, E., Mayr, D., Klauschen, F., Wehweck, F., Rottmann, M., Kessler, M., Kaltofen, T., Czogalla, B., Trillsch, F., Mahner, S., and Schmoeckel, E. (2022) A combination of immunohistochemical markers, MUC1, MUC5AC, PAX8 and growth pattern for characterization of mucinous neoplasm of the ovary. *Int J Gynecol Cancer* **32**, 662-668

88. Schuster-Little, N., Sokolovsky, A. D., Gentry, A., Saraf, A., Etzel, M. R., Patankar, M. S., and Whelan, R. J. (2024) Immunoaffinity-free chromatographic purification of ovarian cancer biomarker CA125 (MUC16) from blood serum enables mass spectrometry characterization. *Anal Methods* **16**, 6337-6348
89. Rey, J. M., Pujol, P., Callier, P., Cavailles, V., Freiss, G., Maudelonde, T., and Brouillet, J. P. (2000) Semiquantitative reverse transcription-polymerase chain reaction to evaluate the expression patterns of genes involved in the oestrogen pathway. *J Mol Endocrinol* **24**, 433-440
90. Havrilesky, L. J., McMahon, C. P., Lobenhofer, E. K., Whitaker, R., Marks, J. R., and Berchuck, A. (2001) Relationship between expression of coactivators and corepressors of hormone receptors and resistance of ovarian cancers to growth regulation by steroid hormones. *J Soc Gynecol Investig* **8**, 104-113
91. Reichenbach, J., Fraungruber, P., Mayr, D., Buschmann, C., Kraus, F. B. T., Topalov, N. E., Chelariu-Raicu, A., Kolben, T., Burges, A., Mahner, S., Kessler, M., Jeschke, U., Czogalla, B., and Trillsch, F. (2023) Nuclear receptor co-repressor NCOR2 and its relation to GPER with prognostic impact in ovarian cancer. *J Cancer Res Clin Oncol* **149**, 8719-8728
92. Hwang, M. H., Cho, K. H., Jeong, K. J., Park, Y. Y., Kim, J. M., Yu, S. L., Park, C. G., Mills, G. B., and Lee, H. Y. (2017) RCP induces Slug expression and cancer cell invasion by stabilizing beta1 integrin. *Oncogene* **36**, 1102-1111
93. Lindsay, A. J., and McCaffrey, M. W. (2017) Rab coupling protein mediated endosomal recycling of N-cadherin influences cell motility. *Oncotarget* **8**, 104717-104732
94. Choe, S. R., Kim, Y. N., Park, C. G., Cho, K. H., Cho, D. Y., and Lee, H. Y. (2018) RCP induces FAK phosphorylation and ovarian cancer cell invasion with inhibition by curcumin. *Exp Mol Med* **50**, 1-10
95. Cho, S. J., Jeong, B. Y., Song, Y. S., Park, C. G., Cho, D. Y., and Lee, H. Y. (2022) STAT3 mediates RCP-induced cancer cell invasion through the NF-kappaB/Slug/MT1-MMP signaling cascade. *Arch Pharm Res* **45**, 460-474
96. Phelan, C. M., Rebbeck, T. R., Weber, B. L., Devilee, P., Ruttledge, M. H., Lynch, H. T., Lenoir, G. M., Stratton, M. R., Easton, D. F., Ponder, B. A., Cannon-

Albright, L., Larsson, C., Goldgar, D. E., and Narod, S. A. (1996) Ovarian cancer risk in BRCA1 carriers is modified by the HRAS1 variable number of tandem repeat (VNTR) locus. *Nat Genet* **12**, 309-311

97. Weitzel, J. N., Ding, S., Larson, G. P., Nelson, R. A., Goodman, A., Grendys, E. C., Ball, H. G., and Krontiris, T. G. (2000) The HRAS1 minisatellite locus and risk of ovarian cancer. *Cancer Res* **60**, 259-261

98. Kar, S. P., Beesley, J., Amin Al Olama, A., Michailidou, K., Tyrer, J., Kote-Jarai, Z., Lawrenson, K., Lindstrom, S., Ramus, S. J., Thompson, D. J., Investigators, A., Kibel, A. S., Dansonka-Mieszkowska, A., Michael, A., Dieffenbach, A. K., Gentry-Maharaj, A., Whittemore, A. S., Wolk, A., Monteiro, A., Peixoto, A., Kierzek, A., Cox, A., Rudolph, A., Gonzalez-Neira, A., Wu, A. H., Lindblom, A., Swerdlow, A., Group, A. S., Australian Cancer, S., BioResource, A., Ziogas, A., Ekici, A. B., Burwinkel, B., Karlan, B. Y., Nordestgaard, B. G., Blomqvist, C., Phelan, C., McLean, C., Pearce, C. L., Vachon, C., Cybulski, C., Slavov, C., Stegmaier, C., Maier, C., Ambrosone, C. B., Hogdall, C. K., Teerlink, C. C., Kang, D., Tessier, D. C., Schaid, D. J., Stram, D. O., Cramer, D. W., Neal, D. E., Eccles, D., Flesch-Janys, D., Edwards, D. R., Wokozorczyk, D., Levine, D. A., Yannoukakos, D., Sawyer, E. J., Bandera, E. V., Poole, E. M., Goode, E. L., Khusnutdinova, E., Hogdall, E., Song, F., Bruinsma, F., Heitz, F., Modugno, F., Hamdy, F. C., Wiklund, F., Giles, G. G., Olsson, H., Wildiers, H., Ulmer, H. U., Pandha, H., Risch, H. A., Darabi, H., Salvesen, H. B., Nevanlinna, H., Gronberg, H., Brenner, H., Brauch, H., Anton-Culver, H., Song, H., Lim, H. Y., McNeish, I., Campbell, I., Vergote, I., Gronwald, J., Lubinski, J., Stanford, J. L., Benitez, J., Doherty, J. A., Permuth, J. B., Chang-Claude, J., Donovan, J. L., Dennis, J., Schildkraut, J. M., Schleutker, J., Hopper, J. L., Kupryjanczyk, J., Park, J. Y., Figueroa, J., Clements, J. A., Knight, J. A., Peto, J., Cunningham, J. M., Pow-Sang, J., Batra, J., Czene, K., Lu, K. H., Herkommer, K., Khaw, K. T., kConFab, I., Matsuo, K., Muir, K., Offitt, K., Chen, K., Moysich, K. B., Aittomaki, K., Odunsi, K., Kiemeney, L. A., Massuger, L. F., Fitzgerald, L. M., Cook, L. S., Cannon-Albright, L., Hooning, M. J., Pike, M. C., Bolla, M. K., Luedeke, M., Teixeira, M. R., Goodman, M. T., Schmidt, M. K., Riggan, M., Aly, M., Rossing, M. A.,

Beckmann, M. W., Moisse, M., Sanderson, M., Southey, M. C., Jones, M., Lush, M., Hildebrandt, M. A., Hou, M. F., Schoemaker, M. J., Garcia-Closas, M., Bogdanova, N., Rahman, N., Investigators, N., Le, N. D., Orr, N., Wentzensen, N., Pashayan, N., Peterlongo, P., Guenel, P., Brennan, P., Paulo, P., Webb, P. M., Broberg, P., Fasching, P. A., Devilee, P., Wang, Q., Cai, Q., Li, Q., Kaneva, R., Butzow, R., Kopperud, R. K., Schmutzler, R. K., Stephenson, R. A., MacInnis, R. J., Hoover, R. N., Winqvist, R., Ness, R., Milne, R. L., Travis, R. C., Benlloch, S., Olson, S. H., McDonnell, S. K., Tworoger, S. S., Maia, S., Berndt, S., Lee, S. C., Teo, S. H., Thibodeau, S. N., Bojesen, S. E., Gapstur, S. M., Kjaer, S. K., Pejovic, T., Tammela, T. L., Network, G., consortium, P., Dork, T., Bruning, T., Wahlfors, T., Key, T. J., Edwards, T. L., Menon, U., Hamann, U., Mitev, V., Kosma, V. M., Setiawan, V. W., Kristensen, V., Arndt, V., Vogel, W., Zheng, W., Sieh, W., Blot, W. J., Kluzniak, W., Shu, X. O., Gao, Y. T., Schumacher, F., Freedman, M. L., Berchuck, A., Dunning, A. M., Simard, J., Haiman, C. A., Spurdle, A., Sellers, T. A., Hunter, D. J., Henderson, B. E., Kraft, P., Chanock, S. J., Couch, F. J., Hall, P., Gayther, S. A., Easton, D. F., Chenevix-Trench, G., Eeles, R., Pharoah, P. D., and Lambrechts, D. (2016) Genome-wide meta-analyses of breast, ovarian, and prostate cancer association studies identify multiple new susceptibility loci shared by at least two cancer types. *Cancer Discov* **6**, 1052-1067

99. Dufresne, J., Bowden, P., Thavarajah, T., Florentinus-Mefailoski, A., Chen, Z. Z., Tucholska, M., Norzin, T., Ho, M. T., Phan, M., Mohamed, N., Ravandi, A., Stanton, E., Slutsky, A. S., Dos Santos, C. C., Romaschin, A., Marshall, J. C., Addison, C., Malone, S., Heyland, D., Scheltens, P., Killestein, J., Teunissen, C. E., Diamandis, E. P., Michael Siu, K. W., and Marshall, J. G. (2018) The plasma peptides of ovarian cancer. *Clin Proteomics* **15**, 41

100. Taliun, D., Harris, D. N., Kessler, M. D., Carlson, J., Szpiech, Z. A., Torres, R., Taliun, S. A. G., Corvelo, A., Gogarten, S. M., Kang, H. M., Pitsillides, A. N., LeFaive, J., Lee, S. B., Tian, X., Browning, B. L., Das, S., Emde, A. K., Clarke, W. E., Loesch, D. P., Shetty, A. C., Blackwell, T. W., Smith, A. V., Wong, Q., Liu, X., Conomos, M. P., Bobo, D. M., Aguet, F., Albert, C., Alonso, A., Ardlie, K. G., Arking, D. E., Aslibekyan, S., Auer, P. L., Barnard, J., Barr, R. G., Barwick, L.,

Becker, L. C., Beer, R. L., Benjamin, E. J., Bielak, L. F., Blangero, J., Boehnke, M., Bowden, D. W., Brody, J. A., Burchard, E. G., Cade, B. E., Casella, J. F., Chalazan, B., Chasman, D. I., Chen, Y. I., Cho, M. H., Choi, S. H., Chung, M. K., Clish, C. B., Correa, A., Curran, J. E., Custer, B., Darbar, D., Daya, M., de Andrade, M., DeMeo, D. L., Dutcher, S. K., Ellinor, P. T., Emery, L. S., Eng, C., Fatkin, D., Fingerlin, T., Forer, L., Fornage, M., Franceschini, N., Fuchsberger, C., Fullerton, S. M., Germer, S., Gladwin, M. T., Gottlieb, D. J., Guo, X., Hall, M. E., He, J., Heard-Costa, N. L., Heckbert, S. R., Irvin, M. R., Johnsen, J. M., Johnson, A. D., Kaplan, R., Kardia, S. L. R., Kelly, T., Kelly, S., Kenny, E. E., Kiel, D. P., Klemmer, R., Konkle, B. A., Kooperberg, C., Kottgen, A., Lange, L. A., Lasky-Su, J., Levy, D., Lin, X., Lin, K. H., Liu, C., Loos, R. J. F., Garman, L., Gerszten, R., Lubitz, S. A., Lunetta, K. L., Mak, A. C. Y., Manichaikul, A., Manning, A. K., Mathias, R. A., McManus, D. D., McGarvey, S. T., Meigs, J. B., Meyers, D. A., Mikulla, J. L., Minear, M. A., Mitchell, B. D., Mohanty, S., Montasser, M. E., Montgomery, C., Morrison, A. C., Murabito, J. M., Natale, A., Natarajan, P., Nelson, S. C., North, K. E., O'Connell, J. R., Palmer, N. D., Pankratz, N., Peloso, G. M., Peyser, P. A., Pleiness, J., Post, W. S., Psaty, B. M., Rao, D. C., Redline, S., Reiner, A. P., Roden, D., Rotter, J. I., Ruczinski, I., Sarnowski, C., Schoenherr, S., Schwartz, D. A., Seo, J. S., Seshadri, S., Sheehan, V. A., Sheu, W. H., Shoemaker, M. B., Smith, N. L., Smith, J. A., Sotoodehnia, N., Stilp, A. M., Tang, W., Taylor, K. D., Telen, M., Thornton, T. A., Tracy, R. P., Van Den Berg, D. J., Vasan, R. S., Viaud-Martinez, K. A., Vrieze, S., Weeks, D. E., Weir, B. S., Weiss, S. T., Weng, L. C., Willer, C. J., Zhang, Y., Zhao, X., Arnett, D. K., Ashley-Koch, A. E., Barnes, K. C., Boerwinkle, E., Gabriel, S., Gibbs, R., Rice, K. M., Rich, S. S., Silverman, E. K., Qasba, P., Gan, W., Consortium, N. T.-O. f. P. M., Papanicolaou, G. J., Nickerson, D. A., Browning, S. R., Zody, M. C., Zollner, S., Wilson, J. G., Cupples, L. A., Laurie, C. C., Jaquish, C. E., Hernandez, R. D., O'Connor, T. D., and Abecasis, G. R. (2021) Sequencing of 53,831 diverse genomes from the NHLBI TOPMed Program. *Nature* **590**, 290-299

101. Feng, P., Ge, Z., Guo, Z., Lin, L., and Yu, Q. (2021) A comprehensive analysis of the downregulation of miRNA-1827 and its prognostic significance by targeting SPTBN2 and BCL2L1 in ovarian cancer. *Front Mol Biosci* **8**, 687576
102. Yang, L., and Gu, Y. (2023) SPTBN2 regulates endometroid ovarian cancer cell proliferation, invasion and migration via ITGB4-mediated focal adhesion and ECM receptor signalling pathway. *Exp Ther Med* **25**, 277
103. Wang, P. H., Lee, W. L., Juang, C. M., Yang, Y. H., Lo, W. H., Lai, C. R., Hsieh, S. L., and Yuan, C. C. (2005) Altered mRNA expressions of sialyltransferases in ovarian cancers. *Gynecol Oncol* **99**, 631-639
104. Fuller, P. J., Zumpe, E. T., Chu, S., Mamers, P., and Burger, H. G. (2002) Inhibin-activin receptor subunit gene expression in ovarian tumors. *J Clin Endocrinol Metab* **87**, 1395-1401
105. Liu, J., Kuulasmaa, T., Kosma, V. M., Butzow, R., Vanttinen, T., Hyden-Granskog, C., and Voutilainen, R. (2003) Expression of betaglycan, an inhibin coreceptor, in normal human ovaries and ovarian sex cord-stromal tumors and its regulation in cultured human granulosa-luteal cells. *J Clin Endocrinol Metab* **88**, 5002-5008
106. Hempel, N., How, T., Dong, M., Murphy, S. K., Fields, T. A., and Blobe, G. C. (2007) Loss of betaglycan expression in ovarian cancer: role in motility and invasion. *Cancer Res* **67**, 5231-5238
107. Bilandzic, M., Chu, S., Farnworth, P. G., Harrison, C., Nicholls, P., Wang, Y., Escalona, R. M., Fuller, P. J., Findlay, J. K., and Stenvers, K. L. (2009) Loss of betaglycan contributes to the malignant properties of human granulosa tumor cells. *Mol Endocrinol* **23**, 539-548
108. Gao, Y., Fang, X., Vincent, D. F., Threadgill, D. W., Bartholin, L., and Li, Q. (2017) Disruption of postnatal folliculogenesis and development of ovarian tumor in a mouse model with aberrant transforming growth factor beta signaling. *Reprod Biol Endocrinol* **15**, 94
109. Choi, A. S., Jenkins-Lane, L. M., Barton, W., Kumari, A., Lancaster, C., Raulerson, C., Ji, H., Altomare, D., Starr, M. D., Whitaker, R., Phaeton, R., Arend, R., Shtutman, M., Nixon, A. B., Hempel, N., Lee, N. Y., and Mythreye, K. (2024) Glycosaminoglycan modifications of betaglycan regulate ectodomain shedding to

fine-tune TGF-beta signaling responses in ovarian cancer. *Cell Commun Signal* **22**, 128

110. Wang, L., and Li, X. (2020) Identification of an energy metabolism-related gene signature in ovarian cancer prognosis. *Oncol Rep* **43**, 1755-1770

111. Romaniuk-Drapala, A., Skupin-Mrugalska, P., Garbuzenko, O., Hatefi, A., and Minko, T. (2024) Synergistic antitumor effect of liposomal-based formulations of olaparib and topotecan in primary epithelial ovarian cancer cells. *Cancer Cell Int* **24**, 285

112. Sun, J., Fang, K., Shen, H., and Qian, Y. (2015) MicroRNA-9 is a ponderable index for the prognosis of human hepatocellular carcinoma. *Int J Clin Exp Med* **8**, 17748-17756

113. Masi, I., Ottavi, F., Del Rio, D., Caprara, V., Vastarelli, C., Giannitelli, S. M., Fianco, G., Mozetic, P., Buttarelli, M., Ferrandina, G., Scambia, G., Gallo, D., Rainer, A., Bagnato, A., Spadaro, F., and Rosano, L. (2023) The interaction of beta-arrestin1 with talin1 driven by endothelin A receptor as a feature of alpha5beta1 integrin activation in high-grade serous ovarian cancer. *Cell Death Dis* **14**, 73

114. Sharbatoghli, M., Saeednejad Zanjani, L., Fahimeh, F., Kalantari, E., Habibi Shams, Z., Panahi, M., Totonchi, M., Asadi-Lari, M., and Madjd, Z. (2023) The association between higher expression of talin-1 and the reduced survival rate in ovarian serous carcinoma patients. *Iran J Pathol* **18**, 312-326

115. Rohozinski, J., Anderson, M. L., Broaddus, R. E., Edwards, C. L., and Bishop, C. E. (2009) Spermatogenesis associated retrogenes are expressed in the human ovary and ovarian cancers. *PLoS One* **4**, e5064

116. Rohozinski, J., Edwards, C. L., and Anderson, M. L. (2012) Does expression of the retrogene UTP14c in the ovary pre-dispose women to ovarian cancer? *Med Hypotheses* **78**, 446-449

117. Lawrenson, K., Song, F., Hazelett, D. J., Kar, S. P., Tyrer, J., Phelan, C. M., Corona, R. I., Rodriguez-Malave, N. I., Seo, J. H., Adler, E., Coetzee, S. G., Segato, F., Fonseca, M. A. S., Amos, C. I., Carney, M. E., Chenevix-Trench, G., Choi, J., Doherty, J. A., Jia, W., Jin, G. J., Kim, B. G., Le, N. D., Lee, J., Li, L.,

Lim, B. K., Adenan, N. A., Mizuno, M., Park, B., Pearce, C. L., Shan, K., Shi, Y., Shu, X. O., Sieh, W., Australian Ovarian Cancer Study, G., Thompson, P. J., Wilkens, L. R., Wei, Q., Woo, Y. L., Yan, L., Karlan, B. Y., Freedman, M. L., Noushmehr, H., Goode, E. L., Berchuck, A., Sellers, T. A., Teo, S. H., Zheng, W., Matsuo, K., Park, S., Chen, K., Pharoah, P. D. P., Gayther, S. A., and Goodman, M. T. (2019) Genome-wide association studies identify susceptibility loci for epithelial ovarian cancer in east Asian women. *Gynecol Oncol* **153**, 343-355

118. Choi, Y. J., Rhee, J. K., Hur, S. Y., Kim, M. S., Lee, S. H., Chung, Y. J., Kim, T. M., and Lee, S. H. (2017) Intraindividual genomic heterogeneity of high-grade serous carcinoma of the ovary and clinical utility of ascitic cancer cells for mutation profiling. *J Pathol* **241**, 57-66

119. Cancer Genome Atlas Research Network. (2011) Integrated genomic analyses of ovarian carcinoma. *Nature* **474**, 609-615

120. Cheasley, D., Wakefield, M. J., Ryland, G. L., Allan, P. E., Alsop, K., Amarasinghe, K. C., Ananda, S., Anglesio, M. S., Au-Yeung, G., Bohm, M., Bowtell, D. D. L., Brand, A., Chenevix-Trench, G., Christie, M., Chiew, Y. E., Churchman, M., DeFazio, A., Demeo, R., Dudley, R., Fairweather, N., Fedele, C. G., Fereday, S., Fox, S. B., Gilks, C. B., Gourley, C., Hacker, N. F., Hadley, A. M., Hendley, J., Ho, G. Y., Hughes, S., Hunstman, D. G., Hunter, S. M., Jobling, T. W., Kalli, K. R., Kaufmann, S. H., Kennedy, C. J., Kobel, M., Le Page, C., Li, J., Lupat, R., McNally, O. M., McAlpine, J. N., Mes-Masson, A. M., Mileshkin, L., Provencher, D. M., Pyman, J., Rahimi, K., Rowley, S. M., Salazar, C., Samimi, G., Saunders, H., Semple, T., Sharma, R., Sharpe, A. J., Stephens, A. N., Thio, N., Torres, M. C., Traficante, N., Xing, Z., Zethoven, M., Antill, Y. C., Scott, C. L., Campbell, I. G., and Gorringe, K. L. (2019) The molecular origin and taxonomy of mucinous ovarian carcinoma. *Nat Commun* **10**, 3935

121. Kandoth, C., McLellan, M. D., Vandin, F., Ye, K., Niu, B., Lu, C., Xie, M., Zhang, Q., McMichael, J. F., Wyczalkowski, M. A., Leiserson, M. D. M., Miller, C. A., Welch, J. S., Walter, M. J., Wendl, M. C., Ley, T. J., Wilson, R. K., Raphael, B. J., and Ding, L. (2013) Mutational landscape and significance across 12 major cancer types. *Nature* **502**, 333-339

122. Lapke, N., Chen, C. H., Chang, T. C., Chao, A., Lu, Y. J., Lai, C. H., Tan, K. T., Chen, H. C., Lu, H. Y., and Chen, S. J. (2021) Genetic alterations and their therapeutic implications in epithelial ovarian cancer. *BMC Cancer* **21**, 499

123. Bashashati, A., Ha, G., Tone, A., Ding, J., Prentice, L. M., Roth, A., Rosner, J., Shumansky, K., Kalloger, S., Senz, J., Yang, W., McConechy, M., Melnyk, N., Anglesio, M., Luk, M. T., Tse, K., Zeng, T., Moore, R., Zhao, Y., Marra, M. A., Gilks, B., Yip, S., Huntsman, D. G., McAlpine, J. N., and Shah, S. P. (2013) Distinct evolutionary trajectories of primary high-grade serous ovarian cancers revealed through spatial mutational profiling. *J Pathol* **231**, 21-34

124. Pi, Y., Sun, F., Zhang, Z., Liu, X., and Lou, G. (2023) A novel Notch-related gene signature for prognosis and immune response prediction in ovarian cancer. *Medicina (Kaunas)* **59**, 1277

125. Hoogstraat, M., de Pagter, M. S., Cirkel, G. A., van Roosmalen, M. J., Harkins, T. T., Duran, K., Kreeftmeijer, J., Renkens, I., Witteveen, P. O., Lee, C. C., Nijman, I. J., Guy, T., van 't Slot, R., Jonges, T. N., Lolkema, M. P., Koudijs, M. J., Zweemer, R. P., Voest, E. E., Cuppen, E., and Kloosterman, W. P. (2014) Genomic and transcriptomic plasticity in treatment-naive ovarian cancer. *Genome Res* **24**, 200-211

126. Abaan, O. D., Polley, E. C., Davis, S. R., Zhu, Y. J., Bilke, S., Walker, R. L., Pineda, M., Gindin, Y., Jiang, Y., Reinhold, W. C., Holbeck, S. L., Simon, R. M., Doroshow, J. H., Pommier, Y., and Meltzer, P. S. (2013) The exomes of the NCI-60 panel: a genomic resource for cancer biology and systems pharmacology. *Cancer Res* **73**, 4372-4382

127. Uhlén, M., Fagerberg, L., Hallström, B. M., Lindskog, C., Oksvold, P., Mardinoglu, A., Sivertsson, A., Kampf, C., Sjostedt, E., Asplund, A., Olsson, I., Edlund, K., Lundberg, E., Navani, S., Szigyarto, C. A., Odeberg, J., Djureinovic, D., Takanen, J. O., Hober, S., Alm, T., Edqvist, P. H., Berling, H., Tegel, H., Mulder, J., Rockberg, J., Nilsson, P., Schwenk, J. M., Hamsten, M., von Feilitzen, K., Forsberg, M., Persson, L., Johansson, F., Zwahlen, M., von Heijne, G., Nielsen, J., and Pontén, F. (2015) Tissue-based map of the human proteome. *Science* **347**, 1260419

128. Yuan, M., Zhang, C., Von Feilitzen, K., Zwahlen, M., Shi, M., Li, X., Yang, H., Song, X., Turkez, H., Uhlén, M., and Mardinoglu, A. (2025) The Human Pathology Atlas for deciphering the prognostic features of human cancers. *EBioMedicine* **111**, 105495

129. McDermott, J. E., Arshad, O. A., Petyuk, V. A., Fu, Y., Gritsenko, M. A., Clauss, T. R., Moore, R. J., Schepmoes, A. A., Zhao, R., Monroe, M. E., Schnaubelt, M., Tsai, C. F., Payne, S. H., Huang, C., Wang, L. B., Foltz, S., Wyczalkowski, M., Wu, Y., Song, E., Brewer, M. A., Thiagarajan, M., Kinsinger, C. R., Robles, A. I., Boja, E. S., Rodriguez, H., Chan, D. W., Zhang, B., Zhang, Z., Ding, L., Smith, R. D., Liu, T., Rodland, K. D., and Clinical Proteomic Tumor Analysis Consortium. (2020) Proteogenomic characterization of ovarian HGSC implicates mitotic kinases, replication stress in observed chromosomal instability. *Cell Rep Med* **1**

130. Hu, Y., Pan, J., Shah, P., Ao, M., Thomas, S. N., Liu, Y., Chen, L., Schnaubelt, M., Clark, D. J., Rodriguez, H., Boja, E. S., Hiltke, T., Kinsinger, C. R., Rodland, K. D., Li, Q. K., Qian, J., Zhang, Z., Chan, D. W., Zhang, H., and Clinical Proteomic Tumor Analysis Consortium. (2020) Integrated proteomic and glycoproteomic characterization of human high-grade serous ovarian carcinoma. *Cell Rep* **33**, 108276
